# Risk Governance of Nanomaterials: Review of Criteria and Tools for Risk Communication, Evaluation, and Mitigation

**DOI:** 10.3390/nano9050696

**Published:** 2019-05-04

**Authors:** Panagiotis Isigonis, Danail Hristozov, Christina Benighaus, Elisa Giubilato, Khara Grieger, Lisa Pizzol, Elena Semenzin, Igor Linkov, Alex Zabeo, Antonio Marcomini

**Affiliations:** 1 Department of Environmental Sciences, Informatics and Statistics, University Ca’ Foscari of Venice, Via Torino 155, 30172 Mestre, Italy; giubilato@unive.it (E.G.); semenzin@unive.it (E.S.); marcom@unive.it (A.M.); 2 GreenDecision s.r.l.—Via delle Industrie, 21/8, 30175 Venice, Italy; lisa.pizzol@greendecision.eu (L.P.); alex.zabeo@greendecision.eu (A.Z.); 3 DIALOGIK—Lerchenstraße 22, 70176 Stuttgart, Germany; benighaus@me.com; 4 Genetic Engineering and Society Center, North Carolina State University, 1070 Partners Way, 5th floor, Raleigh, NC 27695-7565, USA; kdgriege@ncsu.edu; 5 US Army Engineer Research and Development Center, Boston, MA 01472, USA; Igor.Linkov@usace.army.mil; 6 Department of Engineering and Public Policy, College of Engineering, Carnegie Mellon University, Pittsburgh, PA 15213, USA

**Keywords:** manufactured nanomaterials, risk governance, decision analysis, risk communication, risk perception, risk assessment, risk management

## Abstract

Nanotechnologies have been increasingly used in industrial applications and consumer products across several sectors, including construction, transportation, energy, and healthcare. The widespread application of these technologies has raised concerns regarding their environmental, health, societal, and economic impacts. This has led to the investment of enormous resources in Europe and beyond into the development of tools to facilitate the risk assessment and management of nanomaterials, and to inform more robust risk governance process. In this context, several risk governance frameworks have been developed. In our study, we present and review those, and identify a set of criteria and tools for risk evaluation, mitigation, and communication, the implementation of which can inform better risk management decision-making by various stakeholders from e.g., industry, regulators, and the civil society. Based on our analysis, we recommend specific methods from decision science and information technologies that can improve the existing risk governance tools so that they can communicate, evaluate, and mitigate risks more transparently, taking stakeholder perspectives and expert opinion into account, and considering all relevant criteria in establishing the risk-benefit balance of these emerging technologies to enable more robust decisions about the governance of their risks.

## 1. Introduction

Nanotechnologies have been increasingly used in industrial applications and consumer products across several sectors, including construction, transportation, energy, and healthcare. Despite the optimistic projections that manufactured nanomaterials (MNs) can foster technological advancement and contribute to economic growth, there have been growing concerns regarding their possible human health and environmental risks [1,2]. Therefore, to enable responsible and sustainable nanotechnology innovation, it is essential to ensure the adequate governance of these risks, which involves the development and implementation of widely agreed strategies and tools for their prevention, assessment, communication and management [3,4,5]. Risk governance includes the totality of actors, rules, conventions, processes and mechanisms concerned with how relevant risk information is collected, analyzed or communicated and how the risk management decisions are taken [6]. There have been several attempts to formalize this process for emerging technologies in general, and nanotechnology in particular, through the introduction of several risk governance frameworks [5,7,8].

ISO 31000:2009 presents one of the first risk governance frameworks for new technologies, applicable also to MNs. This framework sets principles and provides generic guidelines on risk management to be applied by any organization regardless of its size, activity, or sector. It was updated in 2018 [9] and mainly focused on “risk assessment and treatment”, while “risk communication and monitoring” were identified as important aspects of the risk management decision-making process, but their significance was not thoroughly analyzed. In 2012, the International Risk Governance Council (IRGC) published its risk governance framework specific for MNs, which was updated in 2017 with specific guidelines on governance of emerging risks [10]. Firstly, the IRGC framework focused on the two phases “risk pre-assessment” and “appraisal (assessment)”, where the risks are framed, defined, and governed by societal values, as setting goals and context conditions [11]. Secondly, the framework focused on “characterization and evaluation” and “risk management”, where evidence is collected, and stakeholder judgment is essential for making the necessary trade-offs between risk and benefit [11]. Therefore, communication, stakeholder context, and public engagement serve as cross-cutting aspects of the framework.

Alongside, at European level, the large EU FP7 MARINA, SUN, and NANoREG projects developed frameworks for risk assessment and management of MNs, which together represent a proposal for a common approach for environmental health and safety (EHS) assessment of nanotechnologies consistent with regulations. The SUN and NANoREG frameworks promoted the notion of prevention-based risk governance through Safety-by-Design (SbD). This philosophy was adopted by the NanoReg 2 project, which extended the NANoREG SbD concept [12] to the so-called “safe innovation” approach. This marked the first attempt for transition from risk to innovation governance.

Meanwhile, the EU-funded iNTeg-Risk project developed its Emerging Risk Management Framework (ERMF) [13], which combined and further elaborated elements from the IRGC framework. The ERMF involves “horizon scanning”, “risk pre-assessment”, “risk assessment” and “risk management” processes, complemented by “monitoring and review” and “risk communication” activities. It explicitly addressed nanotechnologies, among other technologies.

The ERMF is being further elaborated in the EU H2020 caLIBRAte project to facilitate risk assessment and management of existing and emerging MNs and nano-enabled products. This is done by testing and calibrating a selection of risk assessment and management tools that match the steps of the “Cooper Stage Gate” [14] product innovation chain and combining them into a System of Systems (SoS) online hub for risk governance of nanotechnologies.

There have been several literature reviews that targeted analytical methodologies for horizon scanning, risk pre-assessment and risk assessment of MNs [7,15,16,17], but although the decision-making and cross-cutting aspects of the risk governance process (i.e., risk evaluation, communication, mitigation) are equally important, the capacity of the existing risk assessment tools to address those has not been comprehensively analyzed. This leaves tool developers with little guidance on how to improve their approaches and/or integrate them into decision-support systems for risk governance. Therefore, to address this gap, the main goals of this review are (i) to assess the capacity of the existing nano-specific risk governance tools to communicate risks in order to effectively inform risk evaluation and management decision-making, based on a set of pre-defined criteria; and (ii) to provide recommendations on methodological developments to fulfil these criteria in order to enable the current and future tools to more effectively support decisions concerned with the risk governance of nanotechnologies.

**Figure 1:**
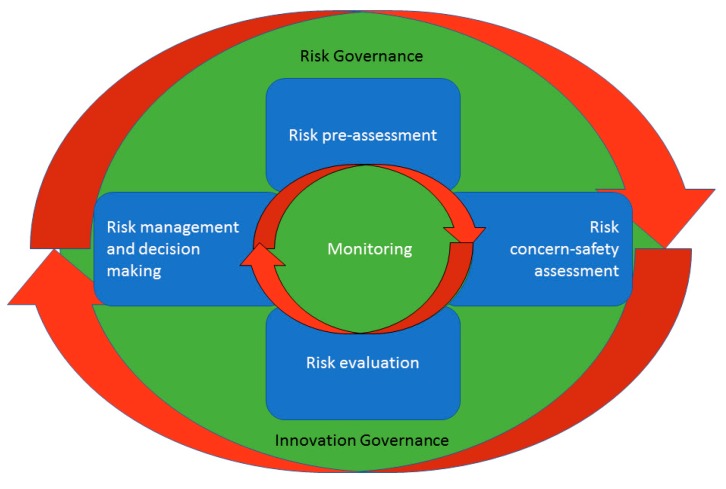
Common elements of the risk governance frameworks for nanomaterials.

For our review, we evaluated and analyzed the existing risk governance frameworks in order to identify their most common elements, and further on, categorize the existing tools for risk governance of nanomaterials within clusters representing those elements, as they can also be seen in Figure 1.

Risk pre-assessment is the stage in which the risk governance processes lead to framing the risk, provide early warning and preparations for handling the risk [10]. Usually pre-assessment involves relevant actors and stakeholder groups, to capture the various perspectives on the risk, its associated opportunities, and potential strategies for addressing it. This phase of the risk governance cycle includes a systematic review of public and stakeholder groups framing their relevant risks topics [11,18].

The IRGC framework includes the notions of risk and concern assessment and safety assessment in the risk appraisal term, which is used for describing the process of assessing the technical and perceived causes and consequences of the risk [10]. This process can be used for developing and synthesizing the knowledge base for the decision on whether a risk should be taken and/or managed or not, and, if so, for identifying and selecting which options may be available for preventing, mitigating, adapting to, or sharing the risk. The appraisal process is dominated by scientific analyses but, in contrast to the traditional risk governance model, the scientific process includes both the natural/technical as well as the social sciences, including economics for producing the best possible scientific estimate of the physical, economic and social consequences of a risk source [11].

Risk evaluation is the process of comparing the outcome of risk appraisal (risk and concern assessment/safety assessment) with specific criteria, to determine the significance and acceptability of the risk, and to formulate decisions [10]. Risk characterization is collecting relevant evidence to make an informed choice of acceptability of the risk, whereas risk evaluation applying societal values and norms to judge the acceptability and the need for risk reduction [11]. Risk characterization and evaluation are therefore linked and are done by risk assessors and risk managers mostly in a joint effort.

Risk management is a process that involves the design and implementation of the actions and remedies required to avoid, reduce (prevent, adapt, mitigate), transfer or retain the risks [10]. Risk management includes the generation, assessment, evaluation, and selection of appropriate management options, the decision about a specific strategy and options, and implementation. It starts with a review from information gathered from the risk appraisal, includes the judgment made in the risk evaluation and formulate different risk management options [11].

Monitoring refers to the evaluation, review, and continuous improvement of the risk governance process.

## 2. Materials and Methods

### 2.1. Identification of Nano-Specific Risk Governance Tools

The peer-reviewed published literature from 1990 to 2018 was searched for journal articles pertaining to the risk governance of nanotechnologies. The Web of Science database was chosen as the main source of information. We performed combined queries with 19 keywords (nano, risk, hazard, exposure, assessment, evaluation, management, governance, perception, communication, framework, methodology, method, model, tool, protocol, database, library, inventory). The search string [TS = (nano* and (risk*) and (hazard*) and (exposure*) and (governance or perception or communication or insurance or assessment or evaluation or management) and (framework or methodology or method or tool or protocol) and (database or library or inventory))] retrieved 883 records, out of which 154 articles have been deemed relevant and contained information on tools related to the risk governance of MNs.

The identified articles have been analyzed for information on tools, methodologies, and frameworks, relevant to the risk communication, evaluation, and mitigation in the context of the risk governance of MNs. The identified literature included review and opinion papers as well as original research articles.

Specific focus has been given to the analysis of results from relevant research projects. To identify those, we performed a search on CORDIS with the same keywords. This revealed several EU-funded projects (e.g., SUN, eNanoMapper, GUIDEnano, NANoREG I/II). The scientific findings from these projects were assessed through a review of their reports, deliverables and toolboxes (such as the NANoREG toolbox [19,20]) that were publicly available or accessible to the authors for the identification of tools, different from the ones derived from our peer-reviewed literature.

Sixty (60) tools have been identified as relevant for our review as a result of the analysis of the overall resources. These tools were grouped according to the common elements of the identified risk governance frameworks (Figure 1).

### 2.2. Identification of Evaluation Criteria and Recommendations to Fulfil the Criteria

The methodology for the identification of the evaluation criteria and recommendations to fulfil those criteria followed a two-step process. Firstly, a dedicated caLIBRAte workshop was organized in March 2017 in Venice, Italy. The workshop was titled “From nano-risk management to innovation governance: Developing state-of-the-art, reliable, and trustable governance models and tools for nanomaterials”. It gathered variety of stakeholders such as technology developers, manufacturers, end users, insurers, researchers, risk managers, regulators, and representatives of civil society organizations (See Table S1 of the Supporting Information). The main objective of the workshop was to engage these stakeholders in discussing their needs and priorities in making decisions concerned with the governance of nanotechnology risks. This contributed to defining criteria for effective risk evaluation, communication, and mitigation both from industrial and regulatory perspectives and ideas on how to fulfil some of those.

To complement the results of the stakeholder workshop, we performed a literature review covering studies published in the period 2008–2018. We reviewed about 40 peer-reviewed articles, book chapters, and reports discussing criteria for evaluation, communication, and mitigation of nanotechnology risks and providing ideas on how to fulfil these criteria. These ideas were thoroughly analyzed by the authors of this manuscript who derived specific recommendations on methodological developments to fulfil these criteria to enable the current and future methods to support more effectively decisions concerned with the risk governance of nanotechnologies. These recommendations are provided in Section 3.3.

## 3. Results

### 3.1. Evaluation Criteria

The methodology described in Section 2.2 resulted in a list of 37 criteria for effective risk evaluation, communication, and mitigation in the context of risk governance of nanotechnologies. These criteria differ considerably in the target and purpose of their use. They include practical communication aspects [21,22,23], evaluate public participation methods [24], apply to risk acceptability and accountability [15,22,24,25,26] or health or environmental risk related aspects [15,16,27]. The 37 identified criteria are not applicable or suitable for assessing all the tools equally, since those tools have been developed for different purposes and in response to different stakeholder needs [16].

Nine (9) criteria have been selected as the most relevant for our analysis and are the highest priority criteria to implement in developing decision-support tools for risk governance. They are presented in Table 1. The evaluation of tools was performed based on those nine (9) criteria, which consider methodological characteristics, such as “uncertainty analysis”, “structured decision-making”, “fair and knowledgeable communication process”, while other address the applicability of the approaches: e.g., “easy to use/understand, user-friendliness”, “quantitative information”, “documented applications/trustworthiness”, “transparency of application/process”, “comprehension” and “influence on final policy”. The selection of each criterion is justified in Table S2 of the Supporting Information while the relevance of the criteria for risk evaluation, mitigation, and communication for each of the different RG stages is presented in Table S3 of the Supporting Information. 

### 3.2. Tools for Risk Governance of MNs

Sixty (60) tools for the risk governance of MNs were identified from the published literature and the research projects and they are grouped into 5 clusters, based on the main components of the risk governance process (Figure 1). The evaluation of each tool against the criteria listed in Table 1 is presented in Table 2, Table 3 and Table 4. Some tools belong to more than one group as they have dual or even multiple purposes. Each of the identified tools is described in the Supporting Information (Tables S5 to S9), while the following sections provide a critical review of the tools against the evaluation criteria.

#### 3.2.1. Risk Pre-Assessment: Early Warning and Screening

Horizon scanning (HS), screening and control-banding are the most common types of tools that fall in the “risk pre-assessment” domain. Twenty-five (25) tools were identified and placed in this category and can be seen in Table 2. Brouwer [31], Hristozov et al. [7] and Liguori et al. [32] comprehensively reviewed the major control-banding and screening tools, whereas Jovanovic et al. [33] reviewed the HS tools. Therefore, we only complement their work by focusing on the risk communication characteristics of the tools and their analysis based on the evaluation criteria.

HS is often used for the identification of emerging issues (or opportunities), such as innovations, associated impacts, risks, and benefits, by scanning the emerging literature (e.g., scientific, peer-reviewed, or otherwise) and then synthesizing this through knowledge management approaches [34]. HS tools generally aim at analyzing automatically existing information from various sources, for the identification of emerging risks, based on various methodologies and data mining techniques. These tools are generally easy to use, they do not incorporate quantitative methodologies or uncertainty analysis techniques but nevertheless can serve as effective means of communication of nanotechnology risks through the transparency of their application and the easy comprehension.

The main HS tools for nanomaterials are the iNTeg-Risk Radar [13] and the Nano-Risk Radar (under development). The first is a tool that identifies and monitor risks, based on environmental, socio-political, economic, regulatory, and technological factors. The latter is an extension of the iNTeg-Risk Radar specifically developed for nanotechnologies within the caLIBRAte project. It is supposed to provide an automatic identification of new nano-specific risks based on internet-based sources and considers cognitive factors in communicating risks in a transparent way to avoid wrong or biased perception of these risks. Tools developed within the project IKnow [35] and the FORCE Intelligent Decision-Support System (IDSS) research project [36] use HS and risk analysis techniques for the anticipation of potential disruptive events and risks from future social and technological trends, respectively. They are non-quantitative tools, useful mainly for information sharing and policy making that could possibly be used for the HS of emerging nanotechnologies. Other horizon scanning tools with similar functionalities in government and industry contexts include the UK Government Horizon Scanning Centre [37] and the “Futurescaper’s HS platform” [38], the Singapore Government Risk Assessment and Horizon Scanning (RAHS) system [37,39], the Cranfield University Horizon Scanning tool [40], the “Swiss Re” SONAR [41] and the annual Allianz’s Risk Barometer [42], which can be used to identify and understand the emerging risks and assist a better preparation for emerging threats, such as MNs in the environment.

Smita et al. [43] developed and applied the “causal diagram assessment” method for nanoparticles, to handle the complex interactions of MNs with environmental processes. It is a non-quantitative methodology that uses available scientific information to describe the interactions, but it also requires extensive knowledge to be applied and interpreted.

Ranking, prioritization and screening tools are (semi-)quantitative tools that use various methodologies and algorithms for analyzing the environmental, human health and occupational hazards or risks of MNs and provide metrics for allowing the ranking of scenarios or risks directly. Their ease of use level varies, as they often require extended expertise for their use, while their results are usually easily understood and communicated.

Hristozov et al. [29,44,45] suggest three of the first MCDA-based tools, which incorporate quantitative techniques and uncertainty analysis for prioritizing occupational risks of MNs, occupational exposure scenarios, and human hazard screening of MNs. The tools require specific/advanced expertise, while they use sound decision-making as well as transparent and well documented processes. Similarly, Tervonen et al. [46] have developed a complex semi-quantitative tool for health and environmental risk classification of MNs based on the “Stochastic multicriteria acceptability analysis (SMAA-TRI)” method. The tool handles uncertainty and provides easily interpreted results. On the other hand, the “Tool for ENM-Application Pair Risk Ranking (TEARR)” [47] is both a quantitative and qualitative tool that is easy to use but does not provide uncertainty analysis.

Screening Tree Tool [48,49,50], NRST (Nanomaterial Risk-Screening Tool) [51] and NanoRiskCat [52,53] are general screening tools that combine hazard and exposure information, with nanomaterial physicochemical properties for ranking MNs and their posing threats as well as aiding risk managers to take decisions under uncertainty. The tools are validated through documented applications, allowing structured decision-making and effective risk communication.

The Nano Guidance for Risk Informed Deployment (NanoGRID) framework developed by the U.S. Army Engineer Research & Development Center (ERDC) [54] is a risk-screening tool in which materials are examined for potential risk in a step-wise manner and with increasing rigor, and limited resources are only allocated to the materials that warrant further scrutiny based on sound scientific reasoning. To perform that, NanoGRID applies a tiered approach to nanomaterial risk (pre-)assessment, which allows identifying when a new technology requires additional risk testing or when it can be addressed within traditional regulatory and safety frameworks. It is a semi-quantitative tool, easy to use but does not offer uncertainty analysis options. It satisfies most criteria as it is well documented, offers a transparent process, it is easy to comprehend, offers structured decision-making, and a fair communication process. Its application could possibly be used to influence policy levels.

Control-Banding (CB) tools represent alternative approaches for risk management based on combined computational hazard and exposure rankings and can be used for the control of the workplace exposure, through the proposal of a range of control measures [32]. The main control-banding tools are CB nanotool [55,56,57], ANSES CB nanotool [58,59], NanoSafer CB tool [60], Stoffenmanager Nano [61] and “Precautionary Matrix for Synthetic Nanomaterials” [62], which are not directly comparable since they have different application domains and are based on different concepts and output formats. All the tools are relatively easy to use and convey efficiently the risk communication messages to the user/stakeholder, with the use of metrics, bands, and general recommendations for actions.

#### 3.2.2. Risk and Concern/Safety Assessment

Our review identified thirty-six (36) tools in this “Risk and concern/safety assessment” category (Table 3). Hristozov et al. [7], Sørensen et al. [63] and Oosterwijk et al. [64] comprehensively reviewed most of such tools for MNs. Therefore, we mainly complement their work by focusing on the risk communication aspects. Sustainable Nanotechnology Decision-Support System (SUNDS) [8,65], GUIDEnano [66], NanoSafer [60], Stoffenmanager Nano [61] and LICARA nanoSCAN [67] are some of the most well-known tools for the assessment of nanomaterials concerns and risks.

SUNDS [8,65] offers a broad set of regulatory risk assessment functionalities, consistent with the REACH requirements, and specifically tailored to MNs. The Decision-Support System (DSS) fulfils our criteria as its design aimed at responding to the needs of various stakeholders not only for risk/safety assessment but also for complete risk governance of MNs. In this context, the tool is quantitative, transparent, takes into consideration uncertainty, is relatively simple to use, is trustworthy, and tested in several industrial case studies. The DSS provides various ways to present the results of the incorporated models, making it easy to comprehend, and is based on a sound decision-making framework. The tool can be considered ideal for risk communication due to its ability to always communicate the magnitude and sources of uncertainty in the risk assessment results in probabilistic terms and by means of easy-to-comprehend charts and figures.

Similarly, GUIDEnano [66] is a tool which has been built with the aim to guide product developers into the design and application of the most appropriate risk assessment and mitigation strategies for a specific nano-enabled product. It is a quantitative tool, easy to use, provides uncertainty analysis options, in a transparent and easy-to-comprehend way. At the moment it is lacking documented applications and offers limited influence on final policy.

NanoSafer [60] is a combined CB and risk assessment tool that enables assessment of the risk level and recommended exposure control associated with production/use of MNs. As an available online tool, it is easy to use, though it requires a significant amount of input data to be used in comparison with other similar tools and does not incorporate uncertainty analysis options. It is quantitative, easy to understand, has documented applications, follows structured decision-making, and supports efficiently policy making and risk communication. Similarly, Stoffenmanager Nano [61] allows the control-banding and risk assessment of MNs through the ranking of potential risks and the proposal of effective risk management measures. Major differences lie on the facts that it is qualitative, does not incorporate uncertainty analysis and the current availability of data may limit its current risk management use. It has similar characteristics with NanoSafer regarding its influence on risk communication.

LICARA nanoSCAN [67] is a very user-friendly and easy-to-comprehend screening-level decision-support tool with relatively low data requirements. It provides a semi-quantitative evaluation of the benefits and risks of MN in products from lifecycle perspective, in a transparent and easy-to-comprehend way while it follows structured decision-making and a fair communication process. It does not offer uncertainty analysis options and may have low influence on final policy.

The Species Sensitivity Distribution (SSD) for nanomaterials [68,69] is a quantitative model for ecological risk evaluation, tailored for nanomaterials. The model is easy to use and documented through case studies. It provides structured information through the use and application of established methodologies, while the results are easily comprehensible and useful for communicating the environmental risks posed by the use of MNs.

REACHnano ToolKit (http://tools.lifereachnano.eu/) is an easy to use, web-based tool, supporting the risk assessment of MNs which offers an inventory of MNs properties and two risk assessment tools for occupational and environmental exposure. It is quantitative and handles instances of uncertainty, while it is transparent, easy to comprehend, and transparent. The following three tools focus on providing access to data inventories or guidance documents therefore our evaluation criteria are not applicable in most instances. The NANEX Exposure Scenario Data Library [28,41] simply contains exposure scenarios of MNs, gathered as part of the NANEX research project. AMBIT2 tool [70] is a quantitative tool that includes a database for nanomaterial properties alongside with advanced data analytics, query and data management modules, and prediction tools. Nano to go! [71] is a Guidance Document that has been prepared within the NanoValid research project and contains information and documents to comprehensively support risk assessment and risk management for the safe handling of MNs. It aims at disseminating and communicating relevant information to researchers and safety experts in a simple and easily comprehensible way.

In the past few years, several models have been developed to assess release and environmental concentrations of MNs, as well as human and environmental exposure and assist the risk assessment of MNs in nano-enabled products. SimpleBox4Nano (SB4N) [72], MendNano [73], NanoDUFLOW [74,75], GWAVA with water quality module [76,77] are environmental fate models for MNs, whereas RedNano [78] is a simulation tool incorporating MendNano with lifecycle impact assessment. Material flow models for MNs include the Stochastic Materials Flow Model [79,80], the Explorative particle flow analysis (PFA) [81,82], the dynamic probabilistic material flow analysis (DP-MFA) [83], and the MFA model 1 [84] and model 2 [85]. A physiologically based pharmacokinetic model (PBPK model) [86] has been developed to model internal human exposure and the Multiple-Path Particle Dosimetry Model (MPPD v 2.11) [87,88] has been developed to model human particle dosimetry. ECETOC TRA v3.1 [89], ConsExpo nano [90], BAUA Sprayexpo 2.3 [91], EGRET [92] are used to model human and environmental exposure to MNs. They all require specific expertise to be run, they usually lack uncertainty analysis functionalities and show intermediate difficulty in their comprehension and understanding by non-experts. Due to their characteristics they are useful for researchers and scientists but provide small contribution to policy making and risk communication to stakeholders or the public.

The Standard Operation Procedure “SOP Tiered Approach for the assessment of exposure to airborne nano-objects in workplaces” [93] covers the overall strategy of assessing exposure to airborne nano-objects in workplaces, following a tiered approach, in 3 hierarchical tiers. The SOP regulates the measurements of exposure in workplaces and influences policy making.

NanoNextNL DSS [94] is a DSS under development with quality-controlled information to aid the risk assessors to prioritize MNs for a full risk assessment and to allow meta-analysis of the available information on (eco)toxicological and exposure data in relation to the measured physicochemical properties of the MNs tested. The tool was not available for review at the time the article was written.

The “Work health and safety assessment tool for handling engineered nanomaterials” [95] aims at assisting stakeholders (organizations or regulators) in risk characterization and the identification of processes and general information related to nanomaterial production and business characteristics. The tool uses a questionnaire that does not require specific expertise to be filled in but is lacking computational characteristics, documented applications, and support for decision-making. FINE (Forecasting the Impacts of Nanomaterials in the Environment) [96,97] is a quantitative model that incorporates nano-specific risk characterization with the use of Bayesian networks and expert elicitation. It is easy to use, allows characterization of risk under uncertainty and has been validated through documented applications. Last, the NanoCommission assessment tool [98] is a questionnaire (available only in German) using assessment criteria to provide classification of MNs or nano-enabled products into cases for concern or not. Since the tool is not available in English, it is likely inaccessible for most users.

In addition, five tools which are used for control-banding and screening provide also concern-assessment functionalities. Specifically, CB nanotool [55,56,57], ANSES CB nanotool [58,59], Swiss Precautionary Matrix [62], NanoRiskCat [52,53] and NanoGRID [54]. NanoGRID tiers 2, 3, and 4 collect quantitative information about environmental releases of MNs, potential ecological exposures based on fate, transport and transformation in the environment, and toxicological impact.

#### 3.2.3. Risk Evaluation (Tolerability/Acceptance)

Our review identified six (6) tools for risk evaluation, all of which belong to the risk assessment (Section 3.2.2) and risk management (Section 3.2.4) categories as well. Results of the evaluation of each tool against the 9 relevant criteria considering the risk evaluation category are reported in the aggregate Table 4, together with the tools identified for the next 2 categories (risk management and monitoring), since the functionalities of these tools mostly overlap and fit multiple sections. These tools are SUNDS [8,65], NanoSafer [60], NanoRiskCat [52,53], REACHnano ToolKit (http://tools.lifereachnano.eu/), LICARA nanoSCAN [67] and NanoGRID [54].

#### 3.2.4. Risk Management—Decision-Making and Support

Our review identified twelve (12) tools that facilitate management and transfer of the health and/or environmental risks from MNs and nano-enabled products, as well as decision-making and support related to those risks (Table 4). SUNDS [8,65] not only addresses nanotechnology risk assessment but if the risks are not properly controlled the system proposes suitable technological alternatives and risk management measures to reduce them to acceptable levels. The tiered structure allows modeling and comparison of scenarios with and without the use of risk management measures, providing essential tools to stakeholders to analyze and communicate the risk related to the analyzed products. The DSS is the only multifunctional, up to this date, risk management DSS that is specifically tailored to MNs.

NanoSafer [60], incorporates a nano-specific hazard-assessment module, which is combined with control-banding paired with risk management recommendations. Similarly, Stoffenmanager Nano [61] proposes a risk-banding tool prioritizing health risks in various scenarios to assist the implementation of control measures. Therefore, they are already reviewed in the respective risk and concern/safety assessment Section of the article (Section 3.2.2).

Nanoinfo.org [78] has been developed as a web-platform to provide access to state-of-the-art resources and tools dedicated to environmental risk assessment and management of MNs. The portal is a collection of databases and quantitative tools and provides access to sound decision-making tools and supports efficiently risk communication. The Nano-specific Risk Management Library (RIVM.nl) has been developed within the NanoReg research project to provide stakeholders with an easy to use tool to select proper RM measures for achieving a high level of protection of the human health and the environment against MNs. In this way, the tool aims at assisting the selection of protection measures and controls in view of limiting the exposure to MNs in the workplace. The tool is qualitative and is limited to providing guidance to the user.

The “low-cost/evidence-based” tool [99] was one of the first efforts to assess and manage the risks associated with MNs (specifically exposure to Carbon Nanofiber) through a validated semi-quantitative model. The tool has limited functionalities compared to the state-of-the-art risk management tools, as it was designed for implementing improvement actions in a manufacturing environment, through a two-tier approach which heavily relied on expert judgment and it was preliminary validated only for a specific nanomaterial and not generally for MNs.

The XL Insurance Database [100,101] was one of the first attempts to adapt LCA focused on MNs and the processes used to manufacture them. The XL Insurance protocol can be used to calculate insurance premiums and is one of the few examples of risk communication and transfer to the insurance industry. The method is semi-quantitative, validated through case studies, and fairly simple to understand.

Within the NANoREG research project, a SbD concept [12] has been developed for MNs in connection with an inventory of existing regulatory accepted toxicity tests applicable for safety screening of MNs [102]. The concept has been extended to the ProSafe SbD Implementation Concept, developed by TEMAS and IPC [103], which further elaborates on the concept of safety dossiers and profiles. Both aim at bridging the gaps between innovators and regulatory authorities by establishing a concept that shares expertise and knowledge between the stakeholders, to help identify uncertainties and potential risks, towards a structured guidance for registration or market approval. They are qualitative but easy to use, offer fair communication as well as transparent and easy-to-comprehend processes.

In addition, four tools which are used for control-banding provide also risk management functionalities. These include CB nanotool [55,56,57], ANSES CB nanotool [58,59], Swiss Precautionary Matrix [62] and NanoRiskCat [52,53].

#### 3.2.5. Monitoring

CENARIOS [104] is the first certifiable nano-specific risk management and monitoring system. CENARIOS supports the risk assessment processes, encompassing risk monitoring tools to introduce specific requirements to responsibly and safely handle MNs. A web-version of the tool is incorporated in SUNDS, as a standalone module, and is implemented through an easy to use questionnaire for supporting decision-making.

### 3.3. Recommendations on Methodological and IT Developments to Fulfil the Identified Criteria in Tools for Risk Governance of Nanotechnologies

Four typologies have been identified as the most relevant areas of methodological and IT development of tools for risk governance of nanotechnologies, with respect to the 9 criteria for risk evaluation, communication and mitigation: i.e., “Decision Analysis”, “Risk Assessment-Management”, “Software Development” and “Statistical Methods”. Eight sectors of development have been identified to help cluster the various techniques based on their characteristics, creating thus one or more sub-areas for each typology, e.g., “Decision Analysis—MCDA methodologies”, “Decision Analysis—Software Development”, “Decision Analysis—Mental modeling” and more. Forty-four (44) methods and techniques have been identified within the (sub-)areas that could be implemented to enable the current and future tools to support risk governance decision more effectively. Those are presented shortly in Table 5, where their relevance for each of our criteria is explained. Interested readers can find further details in Table S4 of the Supporting Information.

## 4. Discussion

Significant advances have been made during the last decade in the field of risk governance of MNs, which have resulted in the development of frameworks for regulating and organizing the risk governance processes in a unified and systematic way. In this work a short analysis of existing risk governance frameworks has been performed, not for analyzing their strengths or weaknesses but for identifying the most important elements and components that are deemed crucial to the risk governance processes and how those are interpreted to specific methodologies and their interconnections in the risk governance paradigm. The risk governance frameworks are used for guidance while their elements drive the processes for early identification and handling of risks, for multiple stakeholder needs, and recommend inclusive approaches to frame, assess, evaluate, manage, and communicate important risk issues, often marked by complexity, uncertainty, and ambiguity.

While the regulatory frameworks have been and continue to be revised to better suit MNs, the supporting models and assessment methods still need to be refined, documented, and taken up by the regulatory system. Moreover, on-going developments and convergence with other enabling technologies will likely pose new and even more complex challenges. Developing and having access to reliable, tailored, and up-to-date tools for evaluation and prioritization of the risks posed by production, use and disposal of MNs, in a context that might imply high uncertainty, is therefore essential for stakeholders involved in taking decisions on these technologies, particularly in the business and insurance sectors [105].

Our study aimed to assess the strengths and limitations of the existing tools for supporting the risk governance of MNs, with a special focus on their suitability for risk communication, evaluation, and mitigation. Risk communication, as well as public and stakeholder engagements, indeed play a crucial role in the cross-cutting aspects of the risk governance frameworks, since open, transparent and inclusive information are very important both for engaging stakeholders to assess and manage risks as well as allowing them to deal with the risk decisions to their respective societal contexts.

We followed a structured methodology for the identification of tools that included the classical review of available literature (peer-reviewed papers) combined with the analysis of research projects and their results, as well as the collection of information from partners and experts in the sector. The identified tools vary from simple questionnaires to databases, advanced models, and complex decision-support platforms. In some instances, they serve multiple purposes and cover more than one of the identified risk governance components, as researchers in the sector develop advanced methodologies and tools, aiming to support and serve multiple risk governance phases in a single environment so that interested stakeholders can use a single tool for fulfilling their needs. Nevertheless, many of the identified tools were built for single specific purposes and their design did not take into consideration a holistic approach of the risk governance cycle, from pre-assessment and risk appraisal, to risk assessment, risk evaluation, and management of MNs. This possibly was due to the rise of production/use of MNs in industry, as part of a fast growing and emerging technology, and the inextricable need to assess and address only specific aspects and sectorial issues as fast as possible. Cross-cutting issues are rarely mentioned in the tools.

Collected feedback from stakeholders within the caLIBRAte project, has shown evidently that the tools for supporting risk governance of MNs should be easy to use and understand, as in many cases the users may lack the expertise to follow complex modeling methodologies or complicated interconnected operations. To this view, the design of tools should take into consideration from the very beginning that there are multiple target audiences which could take advantage of the functionalities of each tool. In the recent years, the use of advanced IT technologies and especially cloud-based platforms has increased. Researchers and developers have shown targeted attention to the satisfaction of stakeholder preferences to design and implement useful and flexible, but at the same time easy to use, tools. In the review, we have identified several tools that satisfy this aspect (e.g., SUNDS, NanoSafer, LICARA, Stoffenmanager Nano and more) and contribute effectively to risk communication in the context of risk governance. At the same time, there is a rise in tools that integrate complicated models into their suite, transformed to fit user-friendly environments, to make possible their use by novice users and provide easy access to complex models to the users and proper guidance on their use.

Most of the reviewed tools are quantitative or semi-quantitative. There is an evident preference in research, industry, regulatory bodies, risk managers and end users for tools which use methodologies that can quantify the notion of risk and provide numeric information for communicating the risk towards the various stakeholders. In many instances, the quantitative tools also incorporate uncertainty analysis capabilities to assess the contribution of variations of model inputs to the assessment results. Clearly communicating the uncertainty and variability in modeling results through sound uncertainty analysis greatly helps decision-making. It could be otherwise easily misled by overconfident communication of uncertain results. If uncertainties are large and deeply embedded, more attention in the communication of results will be needed. In conjunction with these aspects, the reliability of tools and their suitability for risk communication is strongly related to the existence of documented applications. Those are the best ways to test a tool, confirm its functionality, and understand its strengths and limitations. In addition, trustworthiness of input or output sources is important. To this day, most of the tools discussed in this paper have usually only been demonstrated and not thoroughly tested and calibrated, also due to constraints posed by the lack of suitable data to accomplish this task. Data availability and data scarcity are very important issues in the context of risk governance of MNs, as the need for scientific assessments is bounden but the resources to be performed may be insufficient or lacking completely. Tools that require quantitative input information to function cannot be easily applied in data-poor situations, which reduces their overall applicability. In addition, scientific data for MNs should be of demonstrated good quality, validated, reliable, and publicly available to be used in the various risk governance stages. Though the current situation shows that there is great lack of data, there are insufficient frameworks for evaluating the quality of existing data and in many cases the cost/effort to generate new data can be prohibitive. In this way, the calibration and assessment of a tool’s performance becomes difficult. On the other hand, there have been efforts to create tools that aim to systematically gather, share and make publicly available the data related to MNs that exist so far (eNanoMapper, nanoCommons, DaNa 2.0, The nanodatabase, Nanowerk and more) but further efforts are needed to improve their development and exploitation.

The desired positive effects of risk communication would be redundant without transparency in the application/process, which is being followed within a tool and the presence of structured decision-making as part of its design. The latter requires that the applied methodologies should use/provide appropriate mechanisms for structuring and displaying the decision-making process to the user. In this way, a transparent application/process makes it easy for stakeholders to quickly comprehend how specific data points and decision criteria influence the decision-making. The combination of the two elements allows stakeholders to see what is going on and how decisions are being made, and thus comprehend, the risk governance elements and enables them to share the related risk information with third parties. Successful message comprehension by the expected audience is an important aspect of effective risk communication, since stakeholders transmit risk related information to different interested groups, but whether the information is understandable for the target audience or not remains an open question that depends on multiple factors. Simple pre-assessment tools easily satisfy this criterion but the deeper we move in the risk governance processes, the harder it gets to extract and successfully convey the contents of the communication. This is because many of the models/tools require some kind of specific, sectorial expertise, not only for an effective application but for understanding and interpreting their results.

Lastly, regardless on which element of the risk governance framework a tool is focusing on (i.e., risk pre-assessment, risk/safety assessment, risk evaluation, risk management or monitoring), it turned out to be highly important that the output of the applied procedure should be connected to policy making and regulatory purposes and have, even partially, a genuine impact to innovation policy [105]. In other words, tool selection for material assessment for any stage of risk governance must be tailored to the political and institutional requirements of the government(s) that it must operate within. This remains an open question for most of the reviewed tools, as the influence on policy is considered relatively low and mainly unexplored in many disciplines and industrial sectors.

## 5. Conclusions

Based on our analysis, it can be concluded that none of the reviewed tools were able to fulfil 100% all the evaluation criteria that are listed in Table 1, which in our opinion and according to stakeholder consultation should be carefully considered for the development of new tools or the refinement and improvement of the existing ones. Moreover, none of the reviewed tools cover all the components of the risk governance framework and therefore we can conclude that the current availability of tools to support a holistic risk governance of MNs is unsatisfactory. Considering the complexity and multi-faceted and multi-actor nature of risk governance, this conclusion should not surprise but at the same time it highlights the need for further efforts in this field.

As exception, SUNDS, GUIDEnano and NanoSafer emerged as tools which can cover several components of the risk governance framework (namely three: risk assessment, risk evaluation and risk management) and at the same time mostly fulfil the proposed evaluation criteria, contributing most efficiently to risk communication in the context of risk governance. Despite their possible limitations, their features make them appealing for supporting the needs of risk governance of MNs and possible future methodological developments could explore the potential of those tools further. This implies that the tools should allow modular expansion for incorporating tools able to cover the missing risk governance elements.

The attractiveness and efficacy of the risk governance frameworks for stakeholders would be increased with the development of the frameworks into user-friendly web-based decision-support tools, suitable to guide different stakeholder and public groups categories to fulfil the specific requirements and needs of each phase. Therefore, the development of a comprehensive tool for risk governance of MNs is recommended. The tool should integrate all the strengths of the existing tools and couple them in a meaningful and effective way, including new, specific features to improve risk communication efficacy, to support the holistic assessment and facilitate the overall risk governance of MNs. To this end, within the research project caLIBRAte, an effort has been initiated for the development of a System of Systems (SoS) which will serve this purpose and will link different models for risk-screening, control-banding, qualitative and fully integrated predictive quantitative risk assessment, SbD, multicriteria decision-support methods, risk surveillance, risk management, and risk guidance into one framework and tool. Further initiatives in this direction would benefit the implementation of effective risk governance practices and would support safe innovation in all fields of application of nanotechnology.

## Figures and Tables

**Table 1:** Selected relevant criteria for the evaluation of risk governance tools.

Criterion	Description/Justification	Selected References
C1: Uncertainty analysis	Clearly communicating the uncertainty and variability in modeling results through sound uncertainty analysis greatly helps decision-making. It could be otherwise easily misled by overconfident communication of uncertain risk governance results. If uncertainties are large and deeply embedded, more communication will be needed.	[7,15,22,28,29]
C2: Structured decision-making	The participation exercise should use/provide appropriate mechanisms for structuring and displaying the decision-making process.	[24]
C3: Fair and knowledgeable communication process	Accordingly, the scope of risk communication should be broadened to internalize conflicting issues of concern and decision-makers should deepen their analysis to address the embedding of risk issues in value and lifestyle structures.	[23]
C4: Easy to use/understand, user-friendliness	Tools that are easy to use and provide outputs that are easy to assess and do not require specific expertise for their application. Information should be provided clearly to avoid arising misinterpretation. User-friendly tools are particularly relevant for Small and Medium Enterprises (SME) as those companies often do not have staff with experience or specific training suited to apply sophisticated protocols or models and understand the outcomes.	[7,15,26]
C5: Quantitative information	Quantitative tools estimate numerical values for consequences and their probabilities, in specific units defined when developing the context. However, this requires quantitative input information to function and they cannot be easily applied in data-poor situations, which reduces their overall applicability and thus the available risk information that could be communicated to stakeholders.	[7,15,16,26,30]
C6: Documented applications—Trustworthiness	Documented applications are the best way to test a tool, confirm its functionality, and understand its strengths and limitations. Trustworthiness of input or output sources is important.	[7,15,16,30]
C7: Transparency of application—process	To make it easy for stakeholders to quickly comprehend how specific data points and decision criteria influence decision-making. The process should be transparent so that the stakeholders can see what is going on and how decisions are being made.	[7,22,24,26]
C8: Comprehension	Does the audience understand the content of the communication? Often a neglected aspect in the process of communicating the results of risk governance processes, making it hard for stakeholders to exploit the valuable information that is available from the application of the tools.	[21]
C9: Influence on final policy	The output of the procedure should have a genuine impact on policy.	[24]

**Table 2:** Evaluation of the Risk pre-assessment tools, according to 9 relevant criteria. + Criterion fulfilled; − Criterion not fulfilled; ± Criterion not fully fulfilled; NA Criterion not applicable/available.

	Criteria								
Tool	Easy to Use/Understand, User-Friendliness	Quantitative Information	Uncertainty Analysis	Documented Applications/Trustworthiness	Transparency of Application/Process	Comprehension	Influence on Final Policy	Structured Decision-Making	Fair and Knowledgeable Communication Process
iNTeg-Risk Radar	+	−	−	+	+	+	−	NA	+
Nano-Risk Radar	+	−	−	−	+	+	±	+	+
IKnow	+	−	−	−	+	+	±	+	+
FORCE IDSS	+	−	−	−	+	+	−	+	+
UK Gov Horizon Scanning Centre	+	−	−	−	+	+	−	+	+
Futurescaper’s HS platform	+	−	−	−	+	+	−	+	+
RAHS	+	−	−	−	+	+	−	+	+
Cranfield U Horizon Scanning	+	−	−	−	+	+	−	+	+
Swiss Re SONAR	+	−	−	−	+	+	−	+	+
Allianz Risk Barometer	+	−	−	−	+	+	−	+	+
Causal diagram assessment	+	−	−	+	+	±	−	+	+
MCDA procedure for prioritization of Occupational Risks from NMs	-	+	+	+	+	+	−	+	+
MCDA procedure for prioritization of occupational exposure scenarios of NMs	-	+	+	+	+	+	−	+	+
MCDA procedure for hazard screening of ENMs	-	+	+	+	+	+	−	+	+
Stochastic multicriteria acceptability analysis (SMAA-TRI)	-	+	+	+-	+	+	−	+	+
Tool for ENM-Application Pair Risk Ranking (TEARR)	+	±	−	−	+	+	−	+	+
Screening Tree Tool	+	−	−	+	+	+	−	+	+
NRST (Nanomaterial Risk-Screening Tool)	+	+	−	+	+	+	−	+	+
NanoRiskCat	+	−	−	+	+	+	±	+	+
NanoGRID	+	±	−	+	+	+	±	+	+
CB NanoTool	±	−	−	−	+	+	±	+	+
ANSES CB nanotool	±	±	−	+	+	+	±	+	+
NanoSafer CB Tool	+	+	−	+	+	+	±	+	+
Stoffenmanager Nano	±	−	−	+	+	+	±	+	+
Precautionary Matrix	+	−	±	−	±	±	±	+	+

**Table 3:** Evaluation of the Risk and concern/safety assessment tools, according to 9 relevant criteria. + Criterion fulfilled; − Criterion not fulfilled; ± Criterion not fully fulfilled; NA Criterion not applicable/available.

	Criteria								
Tool	Easy to use/Understand, User-friendliness	Quantitative Information	Uncertainty Analysis	Documented Applications/Trustworthiness	Transparency of Application/Process	Comprehension	Influence on Final Policy	Structured Decision-Making	Fair and Knowledgeable Communication Process
SUNDS	+	+	+	+	+	+	±	+	+
GUIDEnano	+	+	+	-	+	+	±	+	+
NanoSafer	+	+	−	+	+	+	±	+	+
Stoffenmanager Nano	±	−	−	+	+	+	±	+	+
LICARA nanoscan	+	±	−	−	+	+	−	+	+
Species Sensitivity Distribution (SSD) for nanomaterials	+	+	−	+	+	+	−	+	+
REACHnano ToolKit	+	+	±	+	+	+	−	−	+
NANEX Exposure Scenario Data Library	+	−	NA	NA	NA	+	−	NA	NA
AMBIT2 tool	±	+	−	−	NA	±	NA	NA	NA
Nano to go!	+	−	NA	NA	NA	+	±	NA	NA
SimpleBox4Nano (SB4N)	−	+	−	+	+	±	−	−	+
MendNano	−	+	−	+	+	±	−	−	+
NanoDUFLOW	−	+	−	+	+	±	−	−	+
GWAVA with water quality module	−	+	−	+	+	±	−	−	+
RedNano	−	+	−	+	+	±	−	−	+
Stochastic Materials Flow Model	−	+	+	+	+	±	−	−	+
Explorative particle flow analysis (PFA)	±	+	+	+	+	±	−	−	+
Dynamic probabilistic material flow model (DP-MFA)	±	+	+	+	+	±	−	−	+
MFA model 1	±	+	−	+	+	±	−	−	+
MFA model 2	−	+	±	+	+	±	−	−	+
PBPK model	−	+	−	+	+	±	−	−	+
Multiple-Path Particle Dosimetry Model (MPPD v 2.11)	−	+	−	+	+	±	−	−	+
ECETOC TRA v3.1	−	+	−	+	+	±	−	−	+
ConsExpo nano	−	+	−	+	+	±	−	−	+
BAUA Sprayexpo 2.3	−	+	−	+	+	±	−	−	+
EGRET2	−	+	−	+	+	±	−	−	+
SOP Tiered Approach for the assessment of exposure to airborne nano-objects in workplaces	±	−	−	−	+	±	+	+	+
NanoNextNL DSS (under development)	NA	NA	NA	NA	NA	NA	NA	NA	NA
Work health and safety assessment tool for handling engineered nanomaterials	+	+	−	−	+	+	−	−	+
FINE (Forecasting the Impacts of Nanomaterials in the Environment)	−	+	±	−	+	+	−	+	+
NanoCommission assessment tool	−	−	−	+	+	−	−	+	+
CB NanoTool	±	−	−	−	+	+	±	+	+
ANSES CB Nanotool	±	±	−	+	+	+	±	+	+
Precautionary Matrix	+	−	±	−	±	±	±	+	+
NanoRiskCat	+	−	−	+	+	+	±	+	+
NanoGRID	+	+-	−	+	+	+	±	+	+

**Table 4:** Evaluation of the Risk evaluation, Risk management–Decision-making and support and Monitoring tools, according to 9 relevant criteria. + Criterion fulfilled; − Criterion not fulfilled; ± Criterion not fully fulfilled; NA Criterion not applicable/available.

	Criteria								
Tool	Easy to Use/Understand, User-Friendliness	Quantitative Information	Uncertainty Analysis	Documented Applications/Trustworthiness	Transparency of Application/Process	Comprehension	Influence on Final Policy	Structured Decision-Making	Fair and Knowledgeable Communication Process
SUNDS	+	+	+	+	+	+	±	+	+
NanoSafer	+	+	−	+	+	+	±	+	+
Stoffenmanager Nano	±	−	−	+	+	+	±	+	+
nanoinfo.org	+	+	−	+	+	+	±	+	+
Nano-specific Risk Management Library	+	-	NA	NA	NA	+	−	NA	NA
Low-cost/evidence-based tool	+	±	−	±	+	+	−	−	+
XL Insurance Database	+	±	−	+	±	±	±	±	+
ProSafe SbD Implementation Concept	+	NA	NA	NA	+	+	±	NA	+
CB NanoTool	±	−	−	−	+	+	±	+	+
ANSES CB Nanotool	±	+	−	+	+	+	±	+	+
Precautionary Matrix	+	−	±	−	±	±	±	+	+
NanoRiskCat	+	−	−	+	+	+	±	+	+
CENARIOS	+	−	−	+	+	+	−	+	+

**Table 5:** Methods and techniques useful to implement the identified criteria in decision-support tools and systems.

#	Typology/Sector	Criteria	Method-Technique-Action and Description
1	Decision Analysis/MCDA methodologies	C1, C2, C3, C4, C5, C6, C7, C8, C9	Multi-Attribute Value Theory (MAVT): MCDA methodology that uses Value (Utility) functions to identify the most preferred alternative or to rank order the alternatives
2	Decision Analysis/MCDA methodologies	C1, C2, C3, C4, C5, C6, C7, C8, C9	Outranking methods: They are based on the concept that an alternative may be dominant, with a certain degree, over another one
3	Decision Analysis/MCDA methodologies	C1, C2, C3, C4, C5, C6, C7, C8, C9	Multi-objective optimization: An area of MCDA concerned with mathematical optimization problems involving more than one objective function to be optimized simultaneously
4	Decision Analysis/MCDA methodologies	C1, C2, C3, C4, C5, C6, C7, C8, C9	Analytic hierarchy process (AHP): MCDA methodology that uses decomposition of the decision problem into a hierarchy of subproblems and evaluation of the relative importance of its various elements by pairwise comparisons
5	Decision Analysis/MCDA methodologies	C1, C2, C3, C4, C5, C6, C7, C8, C9	Fuzzy logic: Introduces a formalization of vagueness and the notion of a degree of satisfaction of an object instead of an absolute evaluation
6	Decision Analysis/MCDA methodologies	C1, C2, C3, C4, C5, C6, C7, C8, C9	Decision trees (decision analysis): A tool to model decisions, outcomes chances, and their possible consequences
7	Decision Analysis/MCDA methodologies	C1, C2, C3, C4, C5, C6, C7, C8, C9	Value of Information (VoI): A methodology that can be used in tiers to explore uncertainty in risk assessment and decision-making
8	Decision Analysis/Mental modeling	C9	Stakeholder profiling/need identification: The process of collecting and reviewing the opinions of relevant stakeholders with respect to the features, capabilities, usability of a decision-support tool
9	Decision Analysis/Mental modeling	C9	Interviews/Focus Groups/Influence diagrams: Different techniques to perform mental modeling methodologies and present results
10	Decision Analysis/Software development	C2, C6, C7	Decision-Support Systems: Building dedicating software for supporting decision-making
11	Risk Assessment-Management/Models	C3, C5	Link-integration of models: Link or integration of various types of models (e.g., ERA-HH-exposure read-across grouping) in a decision-support tool
12	Risk Assessment-Management/Models	C3, C5	Full life cycle/Cooper Stage Gate: Models and tools to cover the full life cycle (ERA, HH, LCIA, Social, EA, Risk Control) and connected to Cooper Stage Gate model. Provide multiple options for the user
13	Risk Assessment-Management/Risk management Measures	C2, C3, C6	Types of Risk Management measures: Link-Integration of RMMs (e.g., Inventory of Technological Alternatives and Risk Management Measures (TARMMs), personalized risk management measures defined by the user or connection to the Exposure Control Efficacy Library (ECEL) database)
14	Risk Assessment-Management/Usability	C1, C2, C3, C4, C5, C7, C8	Automatic conversion system: Introduction of an automatic conversion system, to improve usability of the system
15	Risk Assessment-Management/Usability	C1, C2, C3, C4, C5, C7, C8	Quantal data: Support for quantal data in Human Health Hazard Assessment
16	Risk Assessment-Management/Usability	C1, C2, C3, C4, C5, C7, C8	Nano-specific ontologies: A formal way to describe taxonomies and classification networks, essentially defining the structure of knowledge for various domains, they can be represented and shared through the recognized standard Web Ontology Language
17	Risk Assessment-Management/Usability	C1, C2, C3, C4, C5, C7, C8	Assessment tree interface: Visual flow of sections (tiered approach/connected lifecycle models)
18	Software development/Features	C1, C2, C3, C4, C5, C6, C7, C8, C9	Multiple interfaces: Web application accessible from any web browser, which can also be downloaded and installed in an intranet server. Also supports solutions to the confidentiality issue
19	Software development/Features	C1, C2, C3, C4, C5, C6, C7, C8, C9	Graphical User Interfaces (GUIs): Minimum requirement for modern software-tools
20	Software development/Features	C1, C2, C3, C4, C5, C6, C7, C8, C9	Bugs tracking system: Dedicated system, for efficiently improving Decision-Support Tools
21	Software development/Features	C1, C2, C3, C4, C5, C6, C7, C8, C9	Feature request system: Dedicated system, for efficiently improving Decision-Support Tools
22	Software development/Features	C1, C2, C3, C4, C5, C6, C7, C8, C9	Hosting environment: A crucial component for embedding models in a decision-support tool and allowing smooth operations for the user
23	Software development/Features	C1, C2, C3, C4, C5, C6, C7, C8, C9	Appearance and usability of the web application: Smartly designed applications allow increased user-friendliness and improve risk/uncertainty communication
24	Software development/Features	C1, C2, C3, C4, C5, C6, C7, C8, C9	Public pages: System users can select information for public viewing, allowing communication and partnerships with other stakeholders
25	Software development/Features	C1, C2, C3, C4, C5, C6, C7, C8, C9	Data extraction/migration/interoperability features: Various import, migration, and export features increase user-friendliness of the systems and interoperability
26	Software development/Features	C1, C2, C3, C4, C5, C6, C7, C8, C9	Easy registration/Multiple login methods: Improved usability of a system through multiple ways of identifying users and allowing them to register to the system
27	Software development/Features	C1, C2, C3, C4, C5, C6, C7, C8, C9	Manual/Wiki: User guides in the form of a manual document or documented wiki pages can be used as technical communication documents
28	Software development/Features	C1, C2, C3, C4, C5, C6, C7, C8, C9	Guidance: Interactive guidance of the user to the functionalities of a system
29	Software development/Features	C1, C2, C3, C4, C5, C6, C7, C8, C9	User communication: Systems can use different types of communication protocols for informing users
30	Software development/Features	C1, C2, C3, C4, C5, C6, C7, C8, C9	Case study examples: Documented applications available to the user for experimentation and information sharing
31	Software development/Features	C1, C2, C3, C4, C5, C6, C7, C8, C9	Pairing of functionalities with stakeholder profiling: Driving software developments by implementing identified features through the mental modeling processes
32	Software development/Features	C1, C2, C3, C4, C5, C6, C7, C8, C9	Expandable system (modular): System designed to handle multiple material and needs in the future
33	Software development/Features	C1, C2, C3, C4, C5, C6, C7, C8, C9	Data gaps: Cover lack of data with modeling techniques
34	Software development/Features	C1, C2, C3, C4, C5, C6, C7, C8, C9	API communication: Software to software communication
35	Software development/Features	C1, C2, C3, C4, C5, C6, C7, C8, C9	Type of portal: HUB vs Integrated software
36	Software development/Features	C1, C2, C3, C4, C5, C6, C7, C8, C9	Models: Basic characteristics of models for decision support: Multiple, Fast, Tailored, Embedded, Peer-reviewed, Integrated, Well-known
37	Software development/Features	C1, C2, C3, C4, C5, C6, C7, C8, C9	Public projects: Availability of results to communities
38	Statistical methods/Methodology	C1, C5	Decision Trees (machine learning): A method that uses a tree-like model of decisions and their possible consequences for identifying a strategy most likely to reach a goal
39	Statistical methods/Methodology	C1, C5	Random forests: An ensemble learning method for classification, regression, and other tasks that operates by constructing a multitude of decision trees
40	Statistical methods/Methodology	C1, C5	Sensitivity analysis: Evaluates the effect of changes in input values or assumptions on a model’s results
41	Statistical methods/Methodology	C1, C5	Uncertainty analysis: Investigates the effects of lack of knowledge and other potential sources of error in the model
42	Statistical methods/Methodology	C1, C5	Logistic regression: A predictive regression analysis that can be used to describe data and to explain the relationship between one dependent variable and one or more independent variables
43	Statistical methods/Methodology	C1, C5	Neural networks: An alternative to regression models and other related statistical techniques in the areas of statistical prediction and classification
44	Statistical methods/Methodology	C1, C5	Stable results: Calibration of models to be used in decision-support activities (sensitivity analysis and performance testing)

## Supplementary Materials

The following are available online at https://www.mdpi.com/2079-4991/9/5/696/s1, Table S1: List of Workshop participants, Venice March 2017, Table S2: The complete list of criteria relevant to risk communication for the evaluation of risk governance tools, Table S3: Criteria for risk evaluation, mitigation, and communication relevant for the different phases of the risk governance paradigm, Table S4: Methods and techniques useful to implement the identified criteria in decision-support tools and systems, Table S5: Risk pre-assessment tools descriptions and references, Table S6: Risk concern-assessment tools descriptions and references, Table S7: Risk evaluation tools descriptions and references, Table S8: Risk management decision-making support tools descriptions and references, Table S9: Safety-by-design-monitoring tools descriptions and references.

## References

[1] Maynard A.D., Warheit D.B., Philbert M.A. (2010). The new toxicology of sophisticated materials: Nanotoxicology and beyond. Toxicol. Sci..

[2] Drlickova M., Smolkova B., Runden-Pran E., Dusinska M. (2018). Chapter 6 Health Hazard and Risk Assessment of Nanoparticles Applied in Biomedicine. Nanotoxicology Experimental and Computational Perspectives.

[3] Malloy T., Trump B.D., Linkov I. (2016). Risk-based and prevention-based governance for emerging materials. Environ. Sci. Technol..

[4] Trump B.D., Hristozov D., Malloy T., Linkov I. (2018). Risk associated with engineered nanomaterials: Different tools for different ways to govern. Nano Today.

[5] Stone V., Führ M., Feindt P.H., Bouwmeester H., Linkov I., Sabella S., Murphy F., Bizer K., Tran L., Ågerstrand M. (2018). The Essential Elements of a Risk Governance Framework for Current and Future Nanotechnologies. Risk Anal..

[6] Renn O., Graham P. (2005). Risk Governance—Towards an Integrative Approach.

[7] Hristozov D., Gottardo S., Semenzin E., Oomen A., Bos P., Peijnenburg W., Tongeren M., Nowack B., Hunt N., Brunelli A. (2016). Frameworks and tools for risk assessment of manufactured nanomaterials. Environ. Int..

[8] Subramanian V., Semenzin E., Hristozov D., Zabeo A., Malsch I., McAlea E., Murphy F., Mullins M., van Harmelen T., Ligthart T. (2016). Sustainable nanotechnology decision support system: Bridging risk management, sustainable innovation and risk governance. J. Nanopart. Res..

[9] ISO (2018). ISO 31000:2018—Risk Management—Guidelines.

[10] IRGC (2017). Introduction to the IRGC Risk Governance Framework.

[11] Renn O. (2008). Risk Governance: Coping with Uncertainty in a Complex World.

[12] Noorlander C., Sips A., Hock J., Honeher K., Lehmann H.C. (2016). NANoREG Safe-by-Design (SbD) Concept.

[13] Jovanovic A., Debray B., Ølsen K., Balos D., Batista M., Brouwer D.H., Dien Y., Dolinski K., Duval C., Lopez de Ipina M.J. (2013). Managing Emerging Technology-Related Risks.

[14] Cooper R. (1990). Stage-Gate Systems: A New Tool for Managing New Products. Bus. Horiz..

[15] Hristozov D., Gottardo S., Critto A., Marcomini A. (2012). Risk assessment of engineered nanomaterials: A review of available data and approaches from a regulatory perspective. Nanotoxicology.

[16] Grieger K., Linkov I., Hansen S., Baun A. (2012). Environmental risk analysis for nanomaterials: Review and evaluation of frameworks. Nanotoxicology.

[17] Oomen A.G., Steinhäuser K.G., Bleeker E.A.J., van Broekhuizen F., Sips A., Dekkers S., Wijnhoven S.W.P., Sayre P.G. (2018). Risk assessment frameworks for nanomaterials: Scope, link to regulations, applicability, and outline for future directions in view of needed increase in efficiency. NanoImpact.

[18] Tversky A., Kahneman D., Covello V.T., Mumpower J.L., Stallen P.J.M., Uppuluri V.R.R. (1981). The Framing of Decisions and the Psychology of Choice. Environmental Impact Assessment, Technology Assessment, and Risk Analysis.

[19] Jantunen P., Gottardo S., Crutzen H. (2017). NANoREG Toolbox for the Safety Assessment of Nanomaterials.

[20] Jantunen P., Gottardo S., Rasmussen K., Crutzen H. (2018). An inventory of ready-to-use and publicly available tools for the safety assessment of nanomaterials. NanoImpact.

[21] Weinstein N., Sandman P. (1993). Some Criteria for Evaluating Risk Messages. Risk Anal..

[22] Grobe A., Schneider C., Schetula V., Rekic M., Nawrath S. (2008). Nanotechnologien: Was Verbraucher Wissen Wollen.

[23] Kasperson R. (2014). Four questions for risk communication. J. Risk Res..

[24] Rowe G., Frewer L. (2000). Public Participation Methods: A Framework for Evaluation. Sci. Technol. Hum. Values.

[25] Aven T., Renn O. (2010). Risk Management and Governance Concepts, Guidelines and Applications.

[26] Subramanian V., Semenzin E., Hristozov D., Zondervan-van den Beuken E., Linkov I., Marcomini A. (2015). Review of decision analytic tools for sustainable nanotechnology. Environ. Syst. Decis..

[27] Grobe A., Renn O., Jaeger A., IRGC (2008). Risk Governance of Nanotechnology Applications in Food and Cosmetics.

[28] Stone V., Pozzi-Mucelli S., Tran L., Aschberger K., Sabella S., Vogel U., Poland C., Balharry D., Fernandes T., Gottardo S. (2014). ITS-NANO—Prioritising nanosafety research to develop a stakeholder driven intelligent testing strategy. Part. Fibre Toxicol..

[29] Hristozov D., Zabeo A., Alstrup Jensen K., Gottardo S., Isigonis P., Maccalman L., Critto A., Marcomini A. (2016). Demonstration of a modelling-based multi-criteria decision analysis procedure for prioritisation of occupational risks from manufactured nanomaterials. Nanotoxicology.

[30] Bos P., Gottardo S., Scott-Fordsmand J., van Tongeren M., Semenzin E., Fernandes T., Hristozov D., Hund-Rinke K., Hunt N., Irfan M.A. (2015). The MARINA Risk Assessment Strategy: A Flexible Strategy for Efficient Information Collection and Risk Assessment of Nanomaterials. Int. J. Environ. Res. Public Health.

[31] Brouwer D. (2012). Control Banding Approaches for Nanomaterials. Ann. Occup. Hyg..

[32] Liguori B., Hansen S., Baun A., Jensen K. (2016). Control banding tools for occupational exposure assessment of nanomaterials—Ready for use in a regulatory context?. NanoImpact.

[33] Jovanovic A., Ahmad M., Quintero F.A., Porcari A., Borsella E., Hristozov D., Grieger K., Jensen K. (2017). Comprehensive Analysis of Available Tools and Methodologies for Horizon Scanning.

[34] Owen R., Handy R. (2007). Formulating the problems for environmental risk assessment of nanomaterials. Environ. Sci. Technol..

[35] Raban Y., Remes M., Schroder S. (2015). Final Synthesis Report on Security Oriented Foresight Mapping of Outputs and Methods—Deliverable D3.4. https://cordis.europa.eu/project/rcn/185500/reporting/en.

[36] EU CORDIS (2016). Final Report Summary—FORCE (FOResight Coordination for Europe).

[37] Habegger B. (2009). Horizon Scanning in Government: Concept, Country Experience, and Models for Switzerland.

[38] UK GOVT (2017). Futurescaper Platform.

[39] NSCS (2009). Risk Analysis and Horizon Scanning (RAHS). https://www.nscs.gov.sg/rahs-programme-office.html.

[40] Garnett K., Lickorish F., Rocks S., Prpich G., Rathe A., Pollard S. (2016). Integrating horizon scanning and strategic risk prioritisation using a weight of evidence framework to inform policy decisions. Sci. Total Environ..

[41] Swiss RE (2016). Swiss Re’s SONAR: New Emerging Risk Insight.

[42] Allianz Risk Pulse (2017). Allianz Risk Barometer. https://www.agcs.allianz.com/content/dam/onemarketing/agcs/agcs/reports/Allianz-Risk-Barometer-2017.pdf.

[43] Smita S., Gupta S., Bartonova A., Dusinska M., Gutleb A., Rahman Q. (2012). Nanoparticles in the environment: Assessment using the causal diagram approach. Environ. Health.

[44] Hristozov D., Gottardo S., Cinelli M., Isigonis P., Zabeo A., Critto A., van Tongeren M., Tran L., Marcomini A. (2013). Application of a quantitative weight of evidence approach for ranking and prioritising occupational exposure scenarios for titanium dioxide and carbon nanomaterials. Nanotoxicology.

[45] Hristozov D., Zabeo A., Foran C., Isigonis P., Critto A., Marcomini A., Linkov I. (2014). A weight of evidence approach for hazard screening of engineered nanomaterials. Nanotoxicology.

[46] Tervonen T., Linkov I., Figueira J., Steevens J., Chappell M., Merad M. (2009). Risk-based classification system of nanomaterials. J. Nanopart. Res..

[47] Grieger K., Redmon J., Money E., Widder M., van der Schalie W., Beaulieu S., Womack D. (2015). A relative ranking approach for nano-enabled applications to improve risk-based decision making: A case study of Army materiel. Environ. Syst. Decis..

[48] Askham C. (2011). Environmental Product Development Combining the Life Cycle Perspective with Chemical Hazard Information. Ph.D. Thesis.

[49] Askham C., Gade A., Hanssen O. (2012). Combining REACH, environmental and economic performance indicators for strategic sustainable product development. J. Clean. Prod..

[50] Askham C., Gade A., Hanssen O. (2013). Linking chemical risk information with life cycle assessment in product development. J. Clean. Prod..

[51] Beaudrie C., Kandlikar M., Gregory R., Long G., Wilson T. (2015). Nanomaterial risk screening: A structured approach to aid decision making under uncertainty. Environ. Syst. Decis..

[52] Hansen S., Baun A., Alstrup-Jensen K. (2011). NanoRiskCat—A Conceptual Decision Support Tool for Nanomaterials.

[53] Hansen S., Jensen K., Baun A. (2014). NanoRiskCat: A conceptual tool for categorization and communication of exposure potentials and hazards of nanomaterials in consumer products. J. Nanopart. Res..

[54] Collier Z.A., Kennedy A.J., Poda A.R., Cuddy M.F., Moser R.D., MacCuspie R.I., Harmon A., Plourde K., Haines C.D., Steevens J.A. (2015). Tiered guidance for risk-informed environmental health and safety testing of nanotechnologies. J. Nanopart. Res..

[55] Paik S., Zalk D., Swuste P. (2008). Application of a Pilot Control Banding Tool for Risk Level Assessment and Control of Nanoparticle Exposures. Ann. Occup. Hyg..

[56] Murashov V., Howard J. (2009). Essential features for proactive risk management. Nat. Nanotechnol..

[57] Zalk D., Paik S., Swuste P. (2009). Evaluating the Control Banding Nanotool: A qualitative risk assessment method for controlling nanoparticle exposures. J. Nanopart. Res..

[58] Ostiguy C., Riediker M., Triolet J., Troisfontaines P., Vernez D. (2010). Development of A Specific Control Banding Tool for Nanomaterials.

[59] Riediker M., Ostiguy C., Triolet J., Troisfontaine P., Vernez D., Bourdel G., Thieriet N., Cadène A. (2012). Development of a Control Banding Tool for Nanomaterials. J. Nanomater..

[60] Jensen K., Saber A., Kristensen H., Liguori B., Jensen A., Koponen I., Wallin H. NanoSafer vs. 1.1 Nanomaterial risk assessment using first order modeling. Proceedings of the 6th International Symposium on Nanotechnology, Occupational and Environmental Health.

[61] Van Duuren-Stuurman B., Vink S., Verbist K., Heussen H., Brouwer D., Kroese D., Van Niftrik M.F.J., Tielemans E., Fransman W. (2012). Stoffenmanager Nano Version 1.0: A Web-Based Tool for Risk Prioritization of Airborne Manufactured Nano Objects. Ann. Occup. Hyg..

[62] Höck J., Behra R., Bergamin L., Bourqui-Pittet M., Bosshard C., Epprecht T., Furrer V., Frey S., Gautschi M., Hofmann H. (2018). Guidelines on the Precautionary Matrix for Synthetic Nanomaterials.

[63] Sørensen S.N., Baun A., Burkard M., Dal Maso M., Hansen S.F., Harrison S., Hjorth R., Lofts S., Matzke M., Nowack B. (2019). Evaluating environmental risk assessment models for nanomaterials according to requirements along the product innovation Stage-Gate process. Environ. Sci. Nano.

[64] Oosterwijk T., Stierum R., Franken R., Fransman W., Saamanem A., Kanerva T., Dal Maso M., Poikkimaki M., Jensen K., Liguori B. (2017). Review of Current Hazard, Exposure and (Integrated) HRA Models Considering Their Input Requirements and Applicability at the Cooper Innovation Stage-Gates Defined.

[65] Hristozov D., Pizzol L., Basei G., Zabeo A., Mackevica A., Foss Hansen S., Gosens I., Cassee F.R., de Jong W., Koivisto A.J. (2018). Quantitative human health risk assessment along the lifecycle of nano-scale copper-based wood preservatives. Nanotoxicology.

[66] Park M., Catalán J., Ferraz N., Cabellos J., Vanhauten R., Vázquez-Campos S., Janer G. (2018). Development of a systematic method to assess similarity between nanomaterials for human hazard evaluation purposes—Lessons learnt. Nanotoxicology.

[67] van Harmelen T., Zondervan-van den Beuken E., Brouwer D., Kuijpers E., Fransman W., Buist H., Ligthart T., Hincapié I., Hischier R., Linkov I. (2016). LICARA nanoSCAN—A tool for the self-assessment of benefits and risks of nanoproducts. Environ. Int..

[68] Gottschalk F., Nowack B. (2013). A probabilistic method for species sensitivity distributions taking into account the inherent uncertainty and variability of effects to estimate environmental risk. Integr. Environ. Assess. Manag..

[69] Semenzin E., Lanzellotto E., Hristozov D., Critto A., Zabeo A., Giubilato E., Marcomini A. (2015). Species sensitivity weighted distribution for ecological risk assessment of engineered nanomaterials: The n-TiO_2_ case study. Environ. Toxicol. Chem..

[70] Jeliazkova N., Chomenidis C., Doganis P., Fadeel B., Grafström R., Hardy B., Hastings J., Hegi M., Jeliazkov V., Kochev N. (2015). The eNanoMapper database for nanomaterial safety information. Beilstein J. Nanotechnol..

[71] Niesmann K., Baron M. (2015). ‘Nano to Go’—BAuA’s Latest Contribution to NanoValid.

[72] Meesters J., Koelmans A., Quik J., Hendriks A., van de Meent D. (2014). Multimedia Modeling of Engineered Nanoparticles with SimpleBox4nano: Model Definition and Evaluation. Environ. Sci. Technol..

[73] Liu H., Cohen Y. (2014). Multimedia Environmental Distribution of Engineered Nanomaterials. Environ. Sci. Technol..

[74] Quik J., de Klein J., Koelmans A. (2015). Spatially explicit fate modelling of nanomaterials in natural waters. Water Res..

[75] De Klein J., Quik J., Bäuerlein P., Koelmans A. (2016). Towards validation of the NanoDUFLOW nanoparticle fate model for the river Dommel, The Netherlands. Environ. Sci. Nano.

[76] Dumont E., Williams R., Keller V., Voß A., Tattari S. (2012). Modelling indicators of water security, water pollution and aquatic biodiversity in Europe. Hydrol. Sci. J..

[77] Dumont E., Johnson A., Keller V., Williams R. (2015). Nano silver and nano zinc-oxide in surface waters—Exposure estimation for Europe at high spatial and temporal resolution. Environ. Pollut..

[78] Liu H., Bilal M., Lazareva A., Keller A., Cohen Y. (2015). Simulation tool for assessing the release and environmental distribution of nanomaterials. Beilstein J. Nanotechnol..

[79] Gottschalk F., Scholz R.W., Nowack B. (2010). Probabilistic material flow modeling for assessing the environmental exposure to compounds: Methodology and an application to engineered nano-TiO_2_ particles. Environ. Model. Softw..

[80] Gottschalk F., Sonderer T., Scholz R.W., Nowack B. (2010). Possibilities and limitations of modelling environmental exposure to engineered nanomaterials by probabilistic material flow analysis. Environ. Toxicol. Chem..

[81] Arvidsson R., Molander S., Sandén B. (2011). Impacts of a Silver-Coated Future: Particle Flow Analysis of Silver Nanoparticles. J. Ind. Ecol..

[82] Arvidsson R., Molander S., Sandén B., Hassellöv M. (2011). Challenges in Exposure Modeling of Nanoparticles in Aquatic Environments. Hum. Ecol. Risk Assess. Int. J..

[83] Bornhöft N., Sun T., Hilty L., Nowack B. (2016). A dynamic probabilistic material flow modeling method. Environ. Model. Softw..

[84] Boxall A.B., Chaudhry Q., Sinclair C., Jones A., Aitken R., Jefferson B., Watts C. (2007). Current and Future Predicted Environmental Exposure to Engineered Nanoparticles.

[85] Mueller N., Nowack B. (2008). Exposure Modeling of Engineered Nanoparticles in the Environment. Environ. Sci. Technol..

[86] Riviere J.E., Riviere J.E., Monteiro-Riviere N., Tran C., Vesterdal L. (2014). Pharmacokinetics and Biodistribution of Nanomaterials. Nanotoxicology: Progress toward Nanomedicine.

[87] Anjilvel S., Asgharian B. (1995). A Multiple-Path Model of Particle Deposition in the Rat Lung. Fundam. Appl. Toxicol..

[88] RIVM (2002). Multiple Path Particle Dosimetry Model (MPPD v 1.0): A Model for Human and Rat Airway Particle Dosimetry.

[89] ECETOC (2017). Targeted Risk Assessment (TRA). http://www.ecetoc.org/tools/targeted-risk-assessment-tra/.

[90] Delmaar J.E., van der Zee Park M., van Engelen J.G.M. (2006). ConsExpo—Consumer Exposure and Uptake Models—Program Manual.

[91] Koch W., Behnke W., Berger-Preiß E., Kock H., Gerling S., Hahn S., Schröder K. (2012). BAuA—Repositorium—Validation of An EDP Assisted Model for Assessing Inhalation Exposure and Dermal Exposure during Spraying Processes—Bundesanstalt für Arbeitsschutz und Arbeitsmedizin.

[92] Zaleski R., Qian H., Zelenka M., George-Ares A., Money C. (2014). European solvent industry group generic exposure scenario risk and exposure tool. J. Expo. Sci. Environ. Epidemiol..

[93] Asbach C., Kuhlbusch T.A.J., Kaminski H., Stahlmecke B., Plitzko S., Götz U., Dahmann D. (2012). Standard Operation Procedures for assessing exposure to nanomaterials, following a tiered approach. Nano Gem.

[94] Marvin H., Bouwmeester H., Bakker M., Kroese E., van de Meent D., Bourgeois F., Lokers R., van der Ham H., Verhelst L. (2013). Exploring the development of a decision support system (DSS) to prioritize engineered nanoparticles for risk assessment. J. Nanopart. Res..

[95] Safe Work Australia (2010). Work Health and Safety Assessment Tool for Handling Engineered Nanomaterials.

[96] Money E., Reckhow K., Wiesner M. (2012). The use of Bayesian networks for nanoparticle risk forecasting: Model formulation and baseline evaluation. Sci. Total Environ..

[97] Money E., Barton L., Dawson J., Reckhow K., Wiesner M. (2014). Validation and sensitivity of the FINE Bayesian network for forecasting aquatic exposure to nano-silver. Sci. Total Environ..

[98] Reihlen A., Jepsen D. (2012). Discussion and Results of the German NanoCommission’s Work and the Stakeholder Dialogue “Risk Management in the Nano World”.

[99] Genaidy A., Sequeira R., Rinder M., A-Rehim A. (2009). Risk analysis and protection measures in a carbon nanofiber manufacturing enterprise: An exploratory investigation. Sci. Total Environ..

[100] Sellers K., Sellers K., Sellers J.K., Mackay C., Bergeson L.L., Clough S.R., Hoyt M., Chen J., Henry K. (2009). Balancing the Risks and Rewards. Nanotechnology and the Environment.

[101] Robichaud C.O., Tanzil D., Weilenmann U., Wiesner R.M. (2005). Relative Risk Analysis of Several Manufactured Nanomaterials:  An Insurance Industry Context. Environ. Sci. Technol..

[102] Noorlander C., Bekker C., Soeteman-Hernandez L., Sabella S., Quik J., Peijnenburg W., Prina-Mello A., Sips A. (2016). NANoREG Deliverable D6.4: Inventory of Existing Regulatory Accepted Toxicity Tests Applicable for Safety Screening of MNMs.

[103] TEMAS & IPC (2016). ProSafe Safe-by-Design (SbD) Implementation Concept Final.

[104] Widler T., Meili C., Semenzin E., Subramanian V., Zabeo A., Hristozov D., Marcomini A., Widler T., Meili C., Semenzin E., Subramanian V., Zabeo A., Hristozov D., Marcomini A. (2016). Organisational Risk Management of Nanomaterials Using SUNDS: The Contribution of CENARIOS^®^.

[105] Linkov I., Trump B.D., Anklam E., Berube D., Boisseasu P., Cummings C., Ferson S., Florin M.V., Goldstein B., Hristozov D. (2018). Comparative, collaborative, and integrative risk governance for emerging technologies. Environ. Syst. Decis..

