# Supplementary material for: Risk Governance of Nanomaterials: Review of Criteria and Tools for Risk Communication, Evaluation, and Mitigation
Source: Nanomaterials (Basel). 2019 May 4;9(5):696. doi: 10.3390/nano9050696 (PMC6566360; doi:10.3390/nano9050696)
Supplement: Supplementary file 1 [file nanomaterials-09-00696-s001.pdf]

Supplementary materials

– I – List of Stakeholders – Workshop Venice 2017

The names and complete affiliation of the representatives are not provided for personal data protection and privacy reasons.

Table S1. List of Workshop participants, Venice March 2017.

Delegate	Affiliation	Country
Representative 1	Risk consultant	GERMANY
Representative 2	Risk consultant-Regulator	GERMANY
Representative 3	Academia	ITALY
Representative 4	Manufacturer	ITALY
Representative 5	Academia	ITALY
Representative 6	Research	GERMANY
Representative 7	Research	GERMANY
Representative 8	Civil society organization	ITALY
Representative 9	Risk consultant-Research	FRANCE
Representative 10	Academia	ITALY
Representative 11	Research	ITALY
Representative 12	Academia	FRANCE
Representative 13	Academia	ITALY
Representative 14	Civil society organization	SWITZERLAND
Representative 15	Research	GERMANY
Representative 16	Manufacturer	GERMANY
Representative 17	Academia	ITALY
Representative 18	Research	IRELAND
Representative 19	Academia-Regulator	ITALY
Representative 20	Academia	ITALY
Representative 21	Research	DENMARK
Representative 22	Risk consultant	GERMANY
Representative 23	Regulator	BELGIUM
Representative 24	Manufacturer	BELGIUM
Representative 25	Research	DENMARK
Representative 26	Manufacturer	GERMANY
Representative 27	Regulator	GERMANY
Representative 28	Research	NETHERLANDS
Representative 29	Research	ITALY
Representative 30	Research	GERMANY
Representative 31	Research	FRANCE
Representative 32	Manufacturer	GERMANY
Representative 33	Academia	IRELAND
Representative 34	Research	GERMANY
Representative 35	Academia	ITALY
Representative 36	Research	ITALY
Representative 37	Research	BELGIUM
Representative 38	Risk consultant	GERMANY
Representative 39	Research	ITALY
Representative 40	Insurance	SWITZERLAND
Representative 41	Academia	ITALY
Representative 42	Regulator	BELGIUM

Representative 43	Insurance	FRANCE
Representative 44	Academia	UK
Representative 45	Academia	ITALY
Representative 46	Manufacturer	LUXEMBOURG
Representative 47	Research	NETHERLANDS
Representative 48	Manufacturer	DENMARK
Representative 49	Academia	SWITZERLAND
Representative 50	Academia	ITALY

– II – Evaluation Criteria

Table S2. The complete list of criteria relevant to risk communication for the evaluation of risk governance tools.

Criterion	Description/Justification	Selected references
C1: Uncertainty analysis	Clearly communicating the uncertainty and variability in modeling results through sound uncertainty analysis greatly helps decision-making. It could be otherwise easily misled by overconfident communication of uncertain risk governance results. If uncertainties are large and deeply embedded, more communication will be needed.	[1–5]
C2: Structured decision-making	The participation exercise should use/provide appropriate mechanisms for structuring and displaying the decision-making process.	[6]
C3: Fair and knowledgeable communication process	Accordingly, the scope of risk communication should be broadened to internalize conflicting issues of concern and decision-makers should deepen their analysis to address the embedding of risk issues in value and lifestyle structures.	[3]
C4: Easy to use/understand, user-friendliness	Tools that are easy to use and provide outputs that are easy to analyze, do not require specific expertise for their application. Information should be provided clearly to avoid arising misinterpretation. User-friendly tools are particularly relevant for Small and Medium Enterprises (SME) as those companies often do not have staff with experience or specific training suited to apply sophisticated protocols or models and understand the outcome.	[1,2,7]
C5: Quantitative information	Quantitative tools estimate numerical values for consequences and their probabilities, in specific units defined when developing the context. However, this requires quantitative input information to function and they cannot be easily applied in data-poor situations, which reduces their overall applicability and thus the available risk information that could be communicated to stakeholders.	[1,2,7–9]
C6: Documented applications – Trustworthiness	Documented applications are the best way to test a tool, confirm its functionality and understand its strengths and limitations. Trustworthiness of input or output sources is important.	[1,2,8,9]
C7: Transparency of application/process	To make it easy it is for stakeholders to quickly comprehend how specific data points and decision criteria influence decision-making. The process should be transparent so that the stakeholders can see what is going on and how decisions are being made.	[1,6,7,10]
C8: Comprehension	Does the audience understand the content of the communication?	[11]
C9: Influence on final policy	The output of the procedure should have a genuine impact on policy.	[6]
Effectiveness and efficiency	Effectiveness and efficiency of risk governance processes is desired, especially for a better coordination of dialogues. The information exchange is typical for advanced phases of an inclusive risk debate where in the first phase of risk governance the focus lies on the establishment of a working dialogue and on the recruitment of relevant stakeholders. On the second phase, the call for more effectiveness and efficiency arises due to the different levels of knowledge and expertise of different stakeholders.	[12–14]
Flexible for variety of nanomaterials	Framework or tools should be appropriate for various MNs and variations, to be as comprehensive, robust, and practical as possible.	[8,15–17]
Assessment tier	The assessment tier criterion distinguishes the “screening-level” from the “high-tiers” tools.	[1,4,9]
Lifecycle thinking	It is important to assess the risks of MNs from a lifecycle perspective since the characteristics of some MNs are likely to change significantly during their lifetime, which would affect their hazard, exposure, and risk.	[1,4,9]
Agreement	Does the audience agree with the recommendation or interpretation contained in the message?	[11]
Dose-response consistency	Do people facing a higher dose of a hazard perceive the risk as greater and/or show a greater readiness to act than people exposed to a lower dose of this hazard?	[11]
Hazard-response consistency	Do people facing a hazard that is higher in risk perceive the risk as greater and/or show a greater readiness to act than people exposed to a hazard that is lower in risk?	[11]
Uniformity	Do audience members exposed to the same level of risk tend to have the same responses to this risk?	[11]
Audience evaluation	Does the audience judge the message to have been helpful, accurate, clear, etc.?	[11]
Types of communication failures	When different types of failures are possible, are the failures that occur generally of the more acceptable variety?	[11]
Representativeness of participants	The public participants should comprise a broadly representative sample of the population of the affected public.	[6]
Independence of true participants	The participation process should be conducted in an independent, unbiased way.	[6]
Early involvement	The public should be involved as early as possible in the process as soon as value judgments become salient.	[6]
Resource accessibility	Public participants should have access to the appropriate resources to enable them to successfully fulfil their brief.	[6]
Task definition	The nature and scope of the participation task should be clearly defined	[6]
Cost-effectiveness	The procedure should in some sense be cost-effective.	[6]
Persistence for being effective	Risk communication activities need to be more sustained over time, better funded, and more ambitious in the goals adopted and the outcomes sought.	[3]
Enhancing trust and creating new principles for a democratic outcome	In situations where high social distrust prevails, and this is increasingly common, a thorough revamping of the goals, structure, and conduct of risk communication will be needed.	[3]
Extent of damage	Tools include adverse effects in natural units, such as fatalities, injuries, production losses, etc.	[18]

Probability of occurrence	Estimate/assignment of the relative frequency of an event	[18]
Incertitude	Overall indicator for different uncertainty components	[18]
Ubiquity	Defines the geographic dispersion of potential damages (intra-generational justice)	[18]
Persistency	Tools define the temporal extension of potential damage (intergenerational justice)	[18]
Accountability	Tools enabling trustful relations between the actors and providing the foundation for monitoring and controlling the impacts of risk management outcomes. Accountability implies that claims posed by stakeholders can be substantiated and that scientific results that are brought into the discourse can be validated.	[19–21]
Shared strategic focus	A shared strategic focus is one of the possible structuring and simplifying elements, which are essential for handling complexity, uncertainty, and ambiguity. The term "shared" point to the difference between a societal, dialogue-driven communication approach and a conventional public relations or information strategy. A "shared strategic focus" reflects the experience that stakeholders pursue their own strategies for reaching the required goals. Being strategic is not per se a problem for dialogue.	[19]
Sustainability	General the responsible use of nanotechnologies and nanomaterials, one could claim that this could serve as the lead criterion for a shared strategic focus. The overall aim is to promote innovation in a societal acceptable and legitimate manner so that technological progress is served, and public acceptance and ethical acceptability is enhanced.	[12,19–21]
Politically and legally realizable	The need for the chosen solution to be politically and legally realizable	[12,22]
Ethically and publicly acceptable	The need for chosen solution to be ethically and publicly acceptable	[12,22]

Table S3. Criteria for risk evaluation, mitigation, and communication relevant for the different phases of the risk governance paradigm.

		**Risk Governance phases**				
**#**	**Criteria**	**Risk pre-Assessment**	**Risk Concern Assessment**	**Risk Evaluation**	**Risk Management**	**Monitoring and Communication**
1	Easy to use / understand, user-friendliness	x	x	x	x	x
2	Quantitative information			x	x	x
3	Uncertainty analysis	x	x	x	x	
4	Documented applications / Trustworthiness	x	x	x	x	x
5	Transparency of application / process	x	x	x	x	x
6	Comprehension	x	x	x	x	x
7	Influence on final policy	x	x	x	x	x
8	Structured decision-making	x	x	x	x	x
9	Fair and knowledgeable communication process	x	x	x	x	x
10	Effectiveness and efficiency	x	x	x	x	x
11	Flexible for variety of nanomaterials	x	x	x	x	x
12	Assessment tier	x	x	x	x	x
13	Lifecycle thinking	x	x	x	x	x
14	Agreement					x
15	Dose-response consistency			x	x	x
16	Hazard-response consistency			x	x	x
17	Uniformity					x
18	Audience evaluation	x	x	x	x	x
19	Types of communication failures					x
20	Representativeness of participants	x	x	x	x	x
21	Independence of true participants	x	x	x	x	x
22	Early involvement	x	x	x	x	x
23	Resource accessibility	x	x	x	x	x
24	Task definition	x	x	x	x	x
25	Cost-effectiveness	x	x	x	x	x
26	Persistence for being effective					x
27	Enhancing trust and creating new principles for a democratic outcome					x
28	Extent of damage					x
29	Probability of occurrence					x
30	Incertitude					x
31	Ubiquity					x
32	Persistency					x
33	Accountability					x
34	Shared strategic focus	x	x	x	x	x
35	Sustainability	x	x	x	x	x

36	Politically and legally realizable				x	x
37	Ethically and publicly acceptable				x	x

Table S4. Methods and techniques useful to implement the identified criteria in decision-support tools and systems.

#	Typology / Sector	Criteria	Method-Technique-Action and Description	How the approach can help to fulfil the identified criteria through implementation in decision-support tools
1	Decision Analysis / MCDA methodologies	C1, C2, C3, C4, C5, C6, C7, C8, C9	Multi-Attribute Value Theory (MAVT): MCDA methodology that uses Value (Utility) functions to identify the most preferred alternative or to rank order the alternatives	MCDA methodologies could be used for: 1) examining trade-offs between criteria 2) including user values as preferences for criteria in the decision-making 3) characterizing parameter uncertainty by applying appropriate uncertainty estimation techniques. The choice of methodologies depends on the nature of the desired results and the preferences of the decision maker; therefore it has to be evaluated on a case by case procedure. Overall, the methodologies can be combined and used to support many different tasks simultaneously. They can be applicable to the complete set of criteria or smaller clusters of them
2	Decision Analysis / MCDA methodologies	C1, C2, C3, C4, C5, C6, C7, C8, C9	Outranking methods: They are based on the concept that an alternative may be dominant, with a certain degree, over another one	
3	Decision Analysis / MCDA methodologies	C1, C2, C3, C4, C5, C6, C7, C8, C9	Multi-objective optimization: An area of MCDA concerned with mathematical optimization problems involving more than one objective function to be optimized simultaneously	
4	Decision Analysis / MCDA methodologies	C1, C2, C3, C4, C5, C6, C7, C8, C9	Analytic hierarchy process (AHP): MCDA methodology that uses decomposition of the decision problem into a hierarchy of subproblems and evaluation of the relative importance of its various elements by pairwise comparisons	
5	Decision Analysis / MCDA methodologies	C1, C2, C3, C4, C5, C6, C7, C8, C9	Fuzzy logic: Introduces a formalization of vagueness and the notion of a degree of satisfaction of an object instead of an absolute evaluation	
6	Decision Analysis / MCDA methodologies	C1, C2, C3, C4, C5, C6, C7, C8, C9	Decision trees (decision analysis): A tool to model decisions, outcomes chances, and their possible consequences	Decision trees used in the decision analysis field (as opposed to the machine learning field) can help formal representation of complex decisional cascades. They are a useful tool in structured decision-making
7	Decision Analysis / MCDA methodologies	C1, C2, C3, C4, C5, C6, C7, C8, C9	Value of Information (VoI): A methodology that can be used in tiers to explore uncertainty in risk assessment and decision-making	VoI can help fulfil the uncertainty analysis criterion mainly, as a methodology that can assess which sources of uncertainty can contribute to a reduction of the overall uncertainty in the results of a model
8	Decision Analysis / Mental modeling	C9	Stakeholder profiling/need identification: The process of collecting and reviewing the opinions of relevant stakeholders with respect to the features, capabilities, usability of a decision-support tool	Stakeholder profiling can be used to increase the influence on final policy of decision-support tools by providing in advance specific guidance on the development of decision-support tools. In addition, it supports fulfilling the group of criteria related to audience/participants evaluation and characteristics
9	Decision Analysis / Mental modeling	C9	Interviews / Focus Groups / Influence diagrams: Different techniques to perform mental modeling methodologies and present results	Further to stakeholder profiling, user elicitation and mental modeling techniques can be used for the extraction of information on ease of use/user-friendliness and comprehension
10	Decision Analysis / Software development	C2, C6, C7	Decision-Support Systems: Building dedicating software for supporting decision-making	The design of software tailored to decision-making fulfils the structured decision-making criterion and enhances the criteria transparency of application / documented applications for all the RG phases, while at the same time it supports risk communication in multiple aspects
11	Risk Assessment-Management / Models	C3, C5	Link-integration of models: Link or integration of various types of models (e.g., ERA-HH-exposure read-across grouping) in a decision-support tool	The use of high-quality, peer-reviewed, and well-structured models fulfils the criteria quantitative information, Fair and knowledgeable communication process, effectiveness, and efficiency, Flexible for variety of nanomaterials during the complete RG cycle. Evidently the complexity of the RG paradigm during the life cycle of a nanomaterial from the innovation stage to the end of life can be supported by the use of tailored trustworthy models that can be integrated in the HUB and provide multiple sources of assessment to the users
12	Risk Assessment-Management / Models	C3, C5	Full life cycle / Cooper Stage Gate: Models and tools to cover the full life cycle (ERA, HH, LCIA, Social, EA, Risk Control) and connected to Cooper Stage Gate model. Provide multiple options for the user	The use of models that cover the full life cycle of a material fulfil the criteria assessment tier, lifecycle thinking, and flexible for variety of nanomaterials. They increase user-friendliness and apply to all RG stages

13	Risk Assessment-Management / Risk management Measures	C2, C3, C6	Types of Risk Management measures: Link-Integration of RMMs (e.g., Inventory of Technological Alternatives and Risk Management Measures (TARMMs), personalized risk management measures defined by the user or connection to the Exposure Control Efficacy Library (ECEL) database)	The use of alternatives for integrating risk management measures within a decision-support tool supports the fulfilment of several criteria mainly for the risk management phase of RG by enhancing the risk mitigation and management options (documented applications trustworthiness/structured decision-making/fair and knowledgeable process/flexibility for variety of nanomaterials)
14	Risk Assessment-Management / Usability	C1, C2, C3, C4, C5, C7, C8	Automatic conversion system: Introduction of an automatic conversion system, to improve usability of the system	The use of an automated conversion system increases drastically the ease of use/user-friendliness of a tool and supports multiple criteria through all the RG stages (quantitative information/trustworthiness/transparency/comprehension/knowledgeable communication process/effectiveness)
15	Risk Assessment-Management / Usability	C1, C2, C3, C4, C5, C7, C8	Quantal data: Support for quantal data in Human Health Hazard Assessment	Support of quantal data in HH hazard assessment increases the satisfaction of the quantitative information criterion and enhances the capacity to handle various nanomaterials in the risk assessment phase
16	Risk Assessment-Management / Usability	C1, C2, C3, C4, C5, C7, C8	Nano-specific ontologies: A formal way to describe taxonomies and classification networks, essentially defining the structure of knowledge for various domains, they can be represented and shared through the recognized standard Web Ontology Language (OWL)	Knowledge about nanomaterials, hazard endpoints, compartments etc. needs to be structured in a computer usable way. To this end web ontologies can be used and foster decision analysis as well as application of cascade of tools.
17	Risk Assessment-Management / Usability	C1, C2, C3, C4, C5, C7, C8	Assessment tree interface: Visual flow of sections (tiered approach / connected lifecycle models)	Assessment trees are an excellent way to fulfil the criteria ease of use/comprehension/structured decision-making/transparency of application. They guide the user to a clear visualization of the decision-making process and the process that lies beneath the interface of a decision-support tool
18	Software development / Features	C1, C2, C3, C4, C5, C6, C7, C8, C9	Multiple interfaces: Web application accessible from any web browser, which can also be downloaded and installed in an intranet server. Also supports solutions to the confidentiality issue	Ease of use is satisfied as different users expect different functionalities based on their needs. An important issue from stakeholder profiling is "confidentiality" that can be handled through the use of multiple interfaces
19	Software development / Features	C1, C2, C3, C4, C5, C6, C7, C8, C9	Graphical User Interfaces (GUIs): Minimum requirement for modern software-tools	GUIs increase ease of use and comprehension of a system, for all the RG stages
20	Software development / Features	C1, C2, C3, C4, C5, C6, C7, C8, C9	Bugs tracking system: Dedicated system, for efficiently improving Decision-Support Tools	A specialized tool that enables higher Trustworthiness / Effectiveness and efficiency of a tool for all the RG stages. It enables the possibility to constantly improve an application through testing
21	Software development / Features	C1, C2, C3, C4, C5, C6, C7, C8, C9	Feature request system: Dedicated system, for efficiently improving Decision-Support Tools	A specialized tool that in addition to enabling higher Trustworthiness / Effectiveness and efficiency of a tool for all the RG stages, it supports the fulfilment of criteria structured decision-making/fair and knowledgeable communication process/flexibility for various nanomaterials/assessment tiers/lifecycle thinking by allowing the improvement of an application through user requests
22	Software development / Features	C1, C2, C3, C4, C5, C6, C7, C8, C9	Hosting environment: A crucial component for embedding models in a decision-support tool and allowing smooth operations for the user	Fundamental software development feature that fulfils ease of use / trustworthiness / effectiveness and efficiency of a decision-support tool for all the RG stages
23	Software development / Features	C1, C2, C3, C4, C5, C6, C7, C8, C9	Appearance and usability of the web application: Smartly designed applications allow increased user-friendliness and improve risk/uncertainty communication	An often-neglected characteristic of applications which needs to be taken care of equally during the design and development process and should be considered equally important to theoretical developments of a decision-support tool. It increases user-friendliness and ease of use, as well as lowers the uncertainty on how to use the system and how to communicate results
24	Software development / Features	C1, C2, C3, C4, C5, C6, C7, C8, C9	Public pages: System users can select information for public viewing, allowing communication and partnerships with other stakeholders	Enhances the risk communication stage through sharing of information and fulfilment of criteria such as fair and knowledgeable communication process, documented applications, user-friendliness, and resource accessibility. It also allows the creation of synergies and cooperation between stakeholders
25	Software development / Features	C1, C2, C3, C4, C5, C6, C7, C8, C9	Data extraction/migration/interoperability features: Various import, migration, and export features increase user-friendliness of the systems and interoperability	Effective data handling within a decision-support tool is essential for all the RG stages. A system should allow the user to import-migrate-export data in the easiest and fastest way possible, to improve not only risk assessment and management results but to allow efficient risk communication. Criteria that are fulfilled include ease of use-user-friendliness, documented applications trustworthiness, transparency of application, comprehension, fair and knowledgeable communication process as well as effectiveness and efficiency
26	Software development / Features	C1, C2, C3, C4, C5, C6, C7, C8, C9	Easy registration / Multiple login methods: Improved usability of a system through multiple ways of identifying users and allowing them to register to the system	Ease of use is satisfied as different users expect different functionalities

27	Software development / Features	C1, C2, C3, C4, C5, C6, C7, C8, C9	Manual / Wiki: User guides in the form of a manual document or documented wiki pages can be used as technical communication documents	Breaking down the complexity of a decision-support tool with the use of manuals or wikipages, to increase ease of use/user-friendliness, transparency of application, comprehension, and general risk communication. Such functionalities are important for all the RG stages
28	Software development / Features	C1, C2, C3, C4, C5, C6, C7, C8, C9	Guidance: Interactive guidance of the user to the functionalities of a system	Similarly, to the use of manuals, interactive techniques such as video tutorials provide useful guidance to the user and support similar criteria
29	Software development / Features	C1, C2, C3, C4, C5, C6, C7, C8, C9	User communication: Systems can use different types of communication protocols for informing users	Communication to the users is an essential component of the risk communication stage within RG paradigm. It can be enhanced with the use of multiple ways to present data to users/stakeholders and allow easy comparisons between scenarios, materials, or assessments
30	Software development / Features	C1, C2, C3, C4, C5, C6, C7, C8, C9	Case study examples: Documented applications available to the user for experimentation and information sharing	Supports the documented applications criterion and the trustworthiness of a tool by providing public access to information to the users
31	Software development / Features	C1, C2, C3, C4, C5, C6, C7, C8, C9	Pairing of functionalities with stakeholder profiling: Driving software developments by implementing identified features through the mental modeling processes	Stakeholder profiling can be used to increase the influence on final policy of decision-support tools by providing in advance specific guidance on the development of decision-support tools. In addition, it supports fulfilling the group of criteria related to audience/participants evaluation and characteristics
32	Software development / Features	C1, C2, C3, C4, C5, C6, C7, C8, C9	Expandable system (modular): System designed to handle multiple material and needs in the future	Modular decision-support tools are common in risk decision-making as they support structured decision-making by providing flexibility for tailoring the tools to the needs of users. They improve effectiveness, can allow flexibility for variety of nanomaterials and support tiered assessments for all the RG stages
33	Software development / Features	C1, C2, C3, C4, C5, C6, C7, C8, C9	Data gaps: Cover lack of data with modeling techniques	Data gaps is an important issue in modern RG paradigms. The use of modeling techniques to cover data gaps is a solution to the problem to improve quantitative information and effectiveness of a tool and can mainly be applied to risk appraisal, characterization and management
34	Software development / Features	C1, C2, C3, C4, C5, C6, C7, C8, C9	API communication: Software to software communication	API communication allows software to software communication and is highly important in integrating or linking models to a decision-support tool. Its use can support all the RG stages
35	Software development / Features	C1, C2, C3, C4, C5, C6, C7, C8, C9	Type of portal: HUB vs Integrated software	Both solutions present peculiar advantages, a HUB-based decision-support tool linking all the important information sources and models within a single location makes the acquisition of knowledge faster while the application of models needs more resources. On the opposite side an integrated solution within a single web application requires more initial efforts to grasp the logic in the tool but speeds up models' application by supplying a homogeneous integrated user interface. Holistically, both solutions support the fulfilment of all the criteria for all the RG stages
36	Software development / Features	C1, C2, C3, C4, C5, C6, C7, C8, C9	Models: Basic characteristics of models for decision support: Multiple, Fast, Tailored, Embedded, Peer-reviewed, Integrated, Well-known	Flexibility, assessment tier, and lifecycle thinking are fulfilled by the use of models with the basic characteristics, throughout the RG paradigm
37	Software development / Features	C1, C2, C3, C4, C5, C6, C7, C8, C9	Public projects: Availability of results to communities	Enhances the risk communication stage through sharing of information and fulfilment of criteria such as fair and knowledgeable communication process, documented applications, user-friendliness, and resource accessibility
38	Statistical methods / Methodology	C1, C5	Decision Trees (machine learning): A method that uses a tree-like model of decisions and their possible consequences for identifying a strategy most likely to reach a goal	Decision trees used in the machine learning field (as opposed to the decision analysis field) are useful tools for classification based on learning sets. They are useful in uncertainty analysis, grouping, and the increase of effectiveness and efficiency of decision-support tools
39	Statistical methods / Methodology	C1, C5	Random forests: An ensemble learning method for classification, regression, and other tasks that operate by constructing a multitude of decision trees	Random forests are useful tools for classification based on learning sets. They support uncertainty analysis, grouping, and the increase of the effectiveness and efficiency of decision-support tools
40	Statistical methods / Methodology	C1, C5	Sensitivity analysis: Evaluates the effect of changes in input values or assumptions on a model's results	Sensitivity analysis allows inspection of the stability of results on changes in different inputs and is therefore useful in decisional settings to understand which alternatives are likely to be the most effective
41	Statistical methods / Methodology	C1, C5	Uncertainty analysis: Investigates the effects of lack of knowledge and other potential sources of error in the model	Uncertainty analysis related methods can give an overview of reliability of results and are therefore useful to understand uncertainty itself and its quantification

42	Statistical methods / Methodology	C1, C5	Logistic regression: A predictive regression analysis that can be used to describe data and to explain the relationship between one dependent variable and one or more independent variables	Logistic regression is a tool for binomial classification based on learning sets. It is useful in uncertainty analysis, grouping, and the increase of effectiveness and efficiency of decision-support tools
43	Statistical methods / Methodology	C1, C5	Neural networks: An alternative to regression models and other related statistical techniques in the areas of statistical prediction and classification	Neural networks can be used to predict outcomes of complex nonlinear processes and is therefore useful in decision analysis for grouping or examination of possible alternatives outcomes
44	Statistical methods / Methodology	C1, C5	Stable results: Calibration of models to be used in decision-support activities (sensitivity analysis and performance testing)	The analysis of models through sensitivity analysis and performance testing supports the fulfilment of criteria uncertainty analysis and quantitative information

– III – Description of Identified Tools

Table S5. Risk pre-assessment tools descriptions and references.

Tool Name	Description	References	Sector
NanoRiskRadar	Automatic identification of new risks previously developed for the insurance sector to assess internet-based sources measuring singularity and ubiquity of new information. The tool will also include NM-specific methods to consider cognitive factors (interdependencies between context, objectives and biases) for risk perception.	Under development/caLIBRAte	Scanning
Causal diagram assessment	The causal diagram has been developed as a method to handle the complexity of issues on NP safety, from their exposure to the effects on the environment and health. It gives an overview of available scientific information starting with common sources of NPs and their interactions with various environmental processes that may pose threats to both human health and the environment.	[23]	Scanning
Risk Radar	The RiskRadar uses the social media and widely used internet search streams to predict the trends. The output can be viewed in different visual displays and charts.	[24]	Scanning
IKnow	Identification of Wild Cards (WI) and Weak Signals (WE) in the field of Science, Technology and Innovation (STI).	[25]	Scanning
FORCE	FORCE EU project provides a mapping of past foresight and horizon scanning activities and development of an Intelligent Decision-Support System (IDSS).	[26]	Scanning
Horizon Scanning Centre	The Horizon Scanning Centre is linked to the UK's foresight program and further linked to the government top officials and relevant Ministers. There are two main scans consisting of Delta and Sigma Scans. It is oriented mainly towards public policy.	[27]	Scanning
RAHS	The Risk Assessment and Horizon Scanning (RAHS), as part of National Security Coordination Secretariat (NSCS) explores methods and tools that complement scenario planning in anticipating strategic issues with significant possible impact on Singapore.	[27]	Scanning
Horizon Scanning Cranfield	The Cranfield University has developed a horizon scanning approach, mainly using four types of methods and covers largely 13 key areas that potentially have an impact on the UK.	[28]	Scanning
SONAR	The Swiss Re's SONAR is an internal tool for Swiss Re to scan for early signals related to the emerging risks and trends and inform the Swiss Re's employees about them. Certain information is also shared with external stakeholders.	[29]	Scanning
Risk Barometer	The Allianz's Risk Barometer collate the insights from field experts dispersed in various countries. The top risks are categorized across different regions, countries, industry sectors, and sizes.	[30]	Scanning
Futurescaper's HS platform	The Futurescaper provides software to clients engaged in foresight, scenario planning and other complex strategic issues, especially those involving multiple stakeholders and geographies.	[31]	Scanning
MCDA procedure for prioritization of Occupational Risks from NMs	This paper proposes such a quantitative risk prioritization tool, based on a multicriteria decision analysis algorithm, which combines advanced exposure and dose-response modeling to calculate margins of exposure (MoE) for several MN in order to rank their occupational risks.	[4]	Ranking / priorization
MCDA procedure for hazard screening of ENMs	A quantitative weight of evidence (WOE) framework that uses multicriteria decision analysis methodology for integrating individual studies on nanomaterial hazard resulting from physicochemical and toxicological properties of nanomaterials. The WOE approach explicitly integrates expert evaluation of data quality of available information. Application of the framework is illustrated for titanium dioxide nanoparticles (nano-TiO2), but the approach is designed to compare the relative hazard of several nanomaterials as well as emerging stressors in general.	[32]	Ranking / priorization

MCDA procedure for prioritization of Occupational exposure scenarios of NMs	An approach for relative exposure screening of ENMs. An exposure model explicitly implementing quantitative WOE methods and uses expert judgment for filling data gaps in the available evidence-base. Application of the framework is illustrated for screening of exposure scenarios for nano-scale titanium dioxide, carbon nanotubes, and fullerenes, but it is applicable to other nanomaterials as well.	[33]	Ranking / priorization
Tool for ENM-Application Pair Risk Ranking (TEARR)	This study examines the use of one risk ranking tool that incorporates both quantitative and qualitative information regarding the potential human health risks of ENMs, focused primarily on worker and soldier health. Using a case study involving Army materiel (i.e., equipment), a relative risk ranking algorithm is proposed that accounts for not only the physicochemical characteristics of the ENMs, but also the characteristics of the Army materiel. In this way, the resulting risk potential for soldiers and workers is not solely based on the inherent characteristics of the ENMs but is also influenced within the context of the technology being developed.	[34]	Ranking / priorization
Stochastic multicriteria acceptability analysis (SMAA-TRI)	A decision-support system for classifying nanomaterials into different risk categories. The classification system is based on a set of performance metrics that measure both the toxicity and physicochemical characteristics of the original materials, as well as the expected environmental impacts through the product life cycle. Stochastic multicriteria acceptability analysis (SMAA-TRI), a formal decision analysis method, was used as the foundation for this task. This method allowed us to cluster various nanomaterials in different ecological risk categories based on our current knowledge of nanomaterial physicochemical characteristics, variation in produced material, and best professional judgments. SMAA-TRI uses Monte Carlo simulations to explore all feasible values for weights, criteria measurements, and other model parameters to assess the robustness of nanomaterial grouping for risk management purposes.	[35]	Classification
NRST (Nanomaterial Risk-Screening Tool)	A decision-support framework relating key nanomaterial physicochemical and product characteristics to important hazard and exposure indicators. This framework for aiding risk managers' decisions under uncertainty provides the foundation for the development of a transparent and adaptable screening tool that can inform the management of potential risks.	[36]	Screening
NanoRiskCat	A screening tool that can identify, categorize and rank exposures and effects of nanomaterials used in consumer products based on data available in the peer-reviewed scientific literature and other regulatory relevant sources of information and data. The primary focus was on nanomaterials relevant for professional end users and consumers as, as well as nanomaterials released into the environment. The wider goal of NanoRiskCat is to help manufacturers, downstream end users, regulators, and other stakeholders to evaluate, rank and communicate the potential for exposure and effects through a tiered approach in which the specific applications of a given nanomaterial are evaluated.	[37,38]	Screening
CB NanoTool	The tool estimates an emission probability (without considering exposure controls) and severity band and provides advice on what engineering controls to use. It includes nine domains covering handling of liquids, powders, and abrasion of solids. Combines hazard "severity" and exposure "probability" scores in a matrix to obtain a level of risk and associated controls out of 4 possible levels of increasing risk and associated controls.	[39–41]	Control-banding
Precautionary Matrix for Synthetic Nanomaterials (Swiss Precautionary Matrix)	This tool helps to determine if exposure needs to be controlled, providing advice on whether a precautionary approach is required under normal working conditions, in the worst-case scenario and for the environment.	[42]	Screening
Screening Tree Tool	A screening tool to combine the LCA approach with chemical hazard information (human health and environmental hazard) and exposure pathways. This enabled the product designers to efficiently identify which chemicals and raw materials pose significant hazards and the important exposure pathways. This tool can also be used as a screening tool for new designs/product formulations.	[43–45]	Screening
NanoGRID	Designed to guide users through a tiered testing framework to help characterize the durability, degradation, potential for nano-scale material release and environmental health and safety implications of nano-enabled products.	[46]	Screening
ANSES Nano	The ANSES CB nanotool was developed by the French Agency for Food, Environmental and Occupational Health & Safety (ANSES) to be applied for conducting risk assessment and risk management of work with manufactured nanomaterials or nano-enabled products in industrial settings.	[47,48]	Control-banding

Table S6. Risk concern-assessment tools descriptions and references.

Tool Name	Description	References	Sector
SUNDS	The Sustainable Nanotechnology Decision-Support System (SUNDS) addresses current nanotechnology risk assessment and management needs. The SUNDS conceptual decision framework expands the focus from nanotechnology risk assessment and management to emerging risk governance needs. It has a two-tier structure comprising screening and advanced tools to address varying data availability and stakeholder needs.	[49,50]	Risk assessment
Nanosafer	NanoSafer is a combined control-banding and risk management tool that enables assessment of the risk level and recommended exposure control associated with production and use of manufactured nanomaterials (e.g., nanoparticles, nanoflakes, nanofibers, and nanotubes) in specific work scenarios. In addition to manufactured nanomaterials, the tool can also be used to assess and manage emissions from nanoparticle-forming processes. Uses data on material properties, processes, and production facilities to estimate occupational risk. The tool uses the Risk Quotient (i.e., the ratio of an exposure dose to a human effect threshold) to estimate risk deterministically.<br>The upcoming new version, NanoSafer 2, will be capable of estimating exposure from spray processes. In addition, NanoSafer 2 can perform nano-specific hazard assessment based on read-across between MNs based on specific material properties and hazard indicators, tested for performance against in vivo experiments.	[51]	Risk assessment
GUIDEnano	Assessment and mitigation of nano-enabled product risks on human and environmental health. To develop innovative methodologies to evaluate and manage human and environmental health risks of nano-enabled products, considering the whole product life cycle. Using this tool, industry will be able to evaluate and efficiently mitigate possible health risks for workers, consumers, and the environment associated with the use of nanotechnologies.	[52] - http://www.guidenano.eu/	Risk assessment
ECETOC TRA v3.1	To assess risks associated with nanotechnology operations. Control-banding (CB) strategies (a qualitative risk characterization and management strategy) offer simplified solutions for controlling worker exposures to constituents that are found in the workplace in the absence of firm toxicological and exposure data. Combines hazard "severity" and exposure "probability" scores in a matrix to obtain a level of risk and associated controls out of 4 possible levels of increasing risk and associated controls.	[53]	Risk assessment
LICARA nanoscan	The main goal of LICARA is to develop a structured lifecycle approach for nanomaterials that enables the balance of health/environmental risks of nanomaterials in view of paucity of data against their benefits, and that further allows a comparison with the risks and the benefits of the conventional (non-nano) products. It estimates economic, environmental, and social opportunities. This tool is specifically intended for use by SME to support them in communicating with regulators, and potential clients and investors.	[54]	Risk assessment
EGRET2	ESIG has developed a tool (termed the ESIG GES Risk and Exposure Tool or "EGRET") that enables users to construct their own consumer CSA/ES for a particular area of use within the ESIG/ESVOC library. This library was constructed based on the results of the various communication and use mapping activities that have been undertaken with major Downstream User (DU) groups (e.g., the consumer use of solvents in coatings, which is in turn described by a set of product categories and sub-categories).	[55]	Risk assessment
BAUA Sprayexpo 2.3	SprayExpo is an Excel model for calculation the airborne concentration of the respirable, the thoracic and the inhalable fraction of aerosols containing biocidal substances in indoor environments originating from the release of liquid biocidal sprays.	[56]	Risk assessment
Stoffenmanager Nano	Stoffenmanager Nano allows you to qualitatively assess occupational health risks from inhalation exposure to Manufactured Nano-Objects (MNO). Risk Management Measures may be selected or included in the Action Plan. Stoffenmanager Nano is a "work-in-process" online tool that reflects the current knowledge of risks related to working with nanomaterials.	[57]	Risk assessment
ANSES Nano	The ANSES CB nanotool was developed by the French Agency for Food, Environmental and Occupational Health & Safety (ANSES) to be applied for conducting risk assessment and risk management of work with manufactured nanomaterials or nano-enabled products in industrial settings.	[47,48]	Risk assessment

Control-banding nanotool	Control-banding (CB) strategies offer simplified solutions for controlling worker exposures to constituents that are found in the workplace in the absence of firm toxicological and exposure data. These strategies may be particularly useful in nanotechnology applications, considering the overwhelming level of uncertainty over what nanomaterials present as potential work-related health risks and how these risks can be assessed and managed appropriately. The CB nanotool is a novel CB approach being used at the Lawrence Livermore National Laboratory (LLNL), by both experts and non-experts, to assess risks associated with nanotechnology operations and prescribe appropriate engineering controls. CB nanotool creates a severity and probability risk matrix as an output, which contains four different risk levels.	[39–41]	Risk assessment
Precautionary Matrix for Synthetic Nanomaterials (Swiss Precautionary Matrix)	This tool helps to determine if exposure needs to be controlled, providing advice on whether a precautionary approach is required under normal working conditions, in the worst-case scenario and for the environment.	[42]	Risk assessment
SimpleBox4Nano (SB4N)	Multimedia mass balance model, development of the SimpleBox model. Air, water, soil, sediment compartments. Computes steady state concentrations in all compartments at local, regional, or continental scale. Mechanistic representations of processes. Parameters may be estimated from theory or experiment. Could be applied to dynamic predictions.	[58]	Risk assessment
NanoDUFLOW	Nano enable extension of the DUFLOW hydrological mode. NanoDUFLOW accounts for the ENP transformation processes homo- and hetero-aggregation, dissolution, and degradation, coupled with the transport processes sedimentation, resuspension, and burial to deeper sediment layers. Aggregation and sedimentation are based on Von Smoluchowski and Stokes theories. Aggregation is calculated from the collision frequency for peri- and ortho-kinetic aggregation as well as aggregation due to differential settling, and attachment efficiencies. Hetero-aggregation is modeled for five ENP size classes interacting with five SS size classes leading to 25 classes of hetero-aggregates, all modeled in place and time.	[59,60]	Risk assessment
MendNano	Multimedia mass balance model. Air, water, soil, sediment, biota compartments. Handles size distributions of ENM. Computes concentrations in each compartment over time. Processes: dry and wet deposition to foliage and ground, foliage washoff, aerosolization, wind resuspension, soil-water runoff, hetero-aggregation, dissolution, sedimentation, sediment resuspension, and burial, biotic uptake, and elimination, plant root uptake.	[61]	Risk assessment
RedNano	Integrated simulation tool for assessing the potential release and environmental distribution of nanomaterials based on lifecycle assessment approach and multimedia compartmental modeling coupled with mechanistic intermedia transport processes. The RedNano simulation tool and its web-based software implementation enables scenario analysis to assess the response of an environmental system to various release scenarios. RedNano incorporates the MendNano model.	[62]	Risk assessment
GWAVA with water quality module	Aquatic-only model predicts Predicted Environmental Concentrations (PECs) for river reaches across Europe. Hydrology includes STP discharges, runoff, and water abstraction. Emissions based on per capita NM loadings to sewage and sewage discharge per grid cell. NM transformations modeled via lumped 1st order kinetic loss.	[63,64]	Risk assessment
ConsExpo nano	Tool for the assessment of consumer exposure to nanomaterials via inhalation (spray scenario as well as custom scenarios). The outcome of the assessment is an alveolar load in the lungs as one of the most critical determinants of inflammation of the lungs is both the magnitude and duration of the alveolar load of a nanomaterial. To estimate the alveolar load arising from the use of nano-enabled spray products, ConsExpo nano combines models that estimate the external aerosol concentration in indoor air, with models that estimate the deposition in and clearance of inhaled aerosol from the alveolar region.	[65]- https://www.consexponano.nl/	Risk assessment
Stochastic Materials Flow Model	This model treats input parameters, such as nano-specific production and consumption volumes, fate pathways and transfer coefficients as probability distributions (Monte Carlo, Bayesian and Markov Chain) that are built based on empirical data and expert judgment. Therefore, the outputs of the model are distributions of possible PECs, and its application always includes analysis of variability and uncertainty.	[66,67]	Risk assessment
Dynamic probabilistic material flow model (DP-MFA)	A customized dynamic probabilistic material flow model (DP-MFA) to predict the former, current and future mass-flows of four ENM (nano-TiO2, nano-ZnO, nano-Ag, and CNT) to technical and environmental compartments and the resulting concentrations in these compartments over time.	[68]	Risk assessment

MFA model 1	MFA (Material flow analysis) model for estimating PECs for MNs. Simple system of mathematical algorithms for estimating concentrations in water, soil, and air for a range of exposure scenarios based on data on MN production volumes and uses.	[69]	Risk assessment
MFA model 2	MFA (Material flow analysis) model for estimating PECs for MNs. First model to consider releases of MNs from consumer products in different lifecycle stages; concentrations in air, soil, water, groundwater, and sediments. Certain processes considered important for MNs (aggregation/agglomeration, sedimentation, resuspension, degradation and transformation) not considered in the estimations.	[70]	Risk assessment
Explorative particle flow analysis (PFA)	Dynamic, quantitative environmental fate model based on colloidal chemistry. Estimates particle number concentrations in the aquatic environments resulting from processes such as materials inflow, homo- and hetero-agglomeration/aggregation and sedimentation, which are considered driving forces behind the transport of MN in waters and their potential elimination from them.	[71,72]	Risk assessment
REACHnano ToolKit	A web-based toolkit to support the risk assessment and promote the safe use of NMs along their life cycle. Contains an inventory with information about ca. 30 commonly used NMs. Environmental risk assessment is done through a model flow analysis probabilistic matter (PMFA). The occupational risk assessment tool is based on a combination of control-banding approach, exposure estimation tools, and new templates of exposure scenarios developed specifically for the case of NMs. Users may estimate the exposure depending on the operative conditions and applied risk management measures. Once all the necessary data is introduced, the model estimates if one (or more) scenarios can be dangerous for the worker.	http://tools.lifereachnano.eu/	Risk assessment
NanoRiskCat	A screening tool that can identify, categorize and rank exposures and effects of nanomaterials used in consumer products based on data available in the peer-reviewed scientific literature and other regulatory relevant sources of information and data. The primary focus was on nanomaterials relevant for professional end users and consumers as, as well as nanomaterials released into the environment. The wider goal of NanoRiskCat is to help manufacturers, downstream end users, regulators, and other stakeholders to evaluate, rank and communicate the potential for exposure and effects through a tiered approach in which the specific applications of a given nanomaterial are evaluated.	[37,38]	Risk assessment
PBPK model	A generic physiologically based pharmacokinetic (PBPK) model for nanomaterials, kinetic tool for estimating internal human exposure (post-exposure absorption, distribution and excretion (ADME) of MN). Can be used to characterize the ADME profiles of the MN for a diverse range of species based on particle type and physicochemical properties. Can also help to develop MN-specific uncertainty factors for interspecies differences in kinetics (e.g., between rodents and humans). PBPK modeling may facilitate extrapolation in exposure duration, e.g., tissue concentration levels for chronic exposure. An adaptation and extension of an earlier PBPK model for larger particles, calibrated using data from EU ENPRA, NANOMMUNE, and NANOTEST projects.	[73]	Risk assessment
NANEX Exposure Scenario Data Library	Library of 9 occupational exposure scenarios for a variety of manufactured nanomaterials	[9,29]	Risk assessment
Nano to go!	Guidance document prepared within the EU FP7 NanoValid project for the safe handling of nanomaterials. Contents include a brochure on "Safe handling of nanomaterials and other advanced materials at workplaces" and reports on case studies.	[74] http://www.nanosafetycluster.eu/nanoToGo/	Risk assessment
Multiple-Path Particle Dosimetry Model (MPPD v 2.11)	Particle dosimetry model for airborne particles. The MPPD model is a computational model that can be used for estimating human and rat airway particle dosimetry. The model is applicable to risk assessment, research, and education. The MPPD model calculates the deposition and clearance of monodisperse and polydisperse aerosols in the respiratory tracts of rats and human adults and children (deposition only) for particles ranging in size from ultrafine (0.01 μm) to coarse (20 μm).	[75,76]	Risk assessment
SOP Tiered Approach for the assessment of exposure to airborne nano-objects in workplaces	This SOP covers the overall strategy of assessing exposure to airborne nano-objects in workplaces, following a tiered approach, which contains 3 hierarchical tiers: tier 1: information gathering, tier 2: basic assessment and tier 3: expert assessment. This SOP describes the general procedure, whereas the measurements in tier 2 and tier 3 are described in three main SOPs: Screening, Sampling, and Expanded Measurement. Each of these main SOPs is accompanied by sub-SOPs describing the use of instruments, sample preparation, and data evaluation.	[77]	Risk assessment

AMBIT2 tool	Software tool designed to support companies by facilitating high-quality chemical safety prediction. Based on a "predictive toxicity model", applies the principles of read-across and categorization. AMBIT supports nanomaterials storage (components, physicochemical and biological characterization) and query (connected with eNanoMapper).	[78]	Risk assessment
NanoGRID	Designed to guide users through a tiered testing framework to help characterize the durability, degradation, potential for nano-scale material release and environmental health and safety implications of nano-enabled products.	[46]	Risk assessment
NanoNextNL DSS (under development)	The NanoNextNL DSS aims at helping to identify ENPs and applications that should get priority in the risk assessment.	[79]	Risk assessment

Table S7. Risk evaluation tools descriptions and references.

Tool name	Description	References	Sector
SUNDS	The Sustainable Nanotechnology Decision-Support System (SUNDS) addresses current nanotechnology risk assessment and management needs. The SUNDS conceptual decision framework expands the focus from nanotechnology risk assessment and management to emerging risk governance needs. It has a two-tier structure comprising screening and advanced tools to address varying data availability and stakeholder needs.	[49,50]	Risk characterization
NanoSafer	NanoSafer is a combined control-banding and risk management tool that enables assessment of the risk level and recommended exposure control associated with production and use of manufactured nanomaterials (e.g., nanoparticles, nanoflakes, nanofibers, and nanotubes) in specific work scenarios. In addition to manufactured nanomaterials, the tool can also be used to assess and manage emissions from nanoparticle-forming processes.	[51]	Risk characterization
NanoRiskCat	A screening tool that can identify, categorize and rank exposures and effects of nanomaterials used in consumer products based on data available in the peer-reviewed scientific literature and other regulatory relevant sources of information and data. The primary focus was on nanomaterials relevant for professional end users and consumers as, as well as nanomaterials released into the environment. The wider goal of NanoRiskCat is to help manufacturers, downstream end users, regulators, and other stakeholders to evaluate, rank and communicate the potential for exposure and effects through a tiered approach in which the specific applications of a given nanomaterial are evaluated.	[37,38]	Risk characterization
Species Sensitivity Distribution (SSD) for nanomaterials	A Monte Carlo probabilistic approach is used to generate Species Sensitivity Distribution (SSD) that is then compared with probability distributions of Predicted Environmental Concentrations (PEC) to estimate environmental risks	[80,81]	Risk characterization
Work health and safety assessment tool for handling engineered nanomaterials	A nano-risk assessment tool to assist regulators, research laboratories, and organizations in managing engineered nanomaterials. This tool consists of a questionnaire, which helps to register the chemical composition and the physical form of the nanomaterials manufactured or used, and the safety measures applied to nanoparticle exposure prevention at the workplace.	[82]	Risk characterization
REACHnano ToolKit	A web-based toolkit to support the risk assessment and promote the safe use of NMs along their life cycle. Contains an inventory with information about ca. 30 commonly used NMs. Environmental risk assessment is done through a model flow analysis probabilistic matter (PMFA). The occupational risk assessment tool is based on a combination of control-banding approach, exposure estimation tools, and new templates of exposure scenarios developed specifically for the case of NMs. Users may estimate the exposure depending on the operative conditions and applied risk management measures. Once all the necessary data is introduced, the model estimates if one (or more) scenarios can be dangerous for the worker.	http://tools.lifereachnano.eu/	Risk characterization
nanoinfo.org	A web-platform built to support the nanoinformatics effort by developing and providing state-of-the-art resources and tools dedicated to environmental impact assessment of engineered nanomaterials (ENMs). Consists of:<br>* LearNano: lifecycle assessment of the environmental release of ENMs<br>* MendNano: multimedia compartmental simulation model of the environmental distribution of ENMs<br>* ToxNano: toxicity data analysis of ENMs that supports high-throughput screening and high content studies<br>* NanoEIA: in silico environmental impact analysis platform that enables evaluation of potential impacts and thus can assist in developing risk management options in support of safe-by-design of ENMs considering multicriteria analyses	[62]	Risk characterization
FINE	Baseline probabilistic model that incorporates nano-specific characteristics and environmental parameters, along with elements of exposure potential, hazards, and risks from MN. Bayesian networks in combination with expert elicitation as a tool for nanomaterial risk forecasting.	[83,84]	Risk characterization

LICARA nanoSCAN	Determines and weighs of the benefits and risks over the lifecycle of MN-based products. This tool is specifically intended for use by SME to support them in communicating with regulators, and potential clients and investors. It uses principles and assessment criteria from the Precautionary Matrix, NanoRiskCat and Stoffenmanager Nano, and integrates them with expert judgment through MCDA.	[54]	Risk characterization
NanoCommission assessment tool	A downloadable questionnaire (available only in German). The set of assessment criteria applied to all lifecycle stages are probability of exposure, physicochemical properties, environmental fate and toxicology/ecotoxicology. A similar product not containing nanoparticles is used as a reference. Benefits and risks are considered for consumers, society, environment, and companies at different stages of the lifecycle of a nanomaterial. A classification into two groups is made depending on whether there is cause for concern or not.	[85]	Risk characterization

Table S8. Risk management decision-making support tools descriptions and references.

Tool Name	Description	References	Sector
SUNDS	The Sustainable Nanotechnology Decision-Support System (SUNDS) addresses current nanotechnology risk assessment and management needs. The SUNDS conceptual decision framework expands the locus from nanotechnology risk assessment and management to emerging risk governance needs. It has a two-tier structure comprising screening and advanced tools to address varying data availability and stakeholder needs.	[49,50]	Risk management
CB Nanotool	The tool estimates an emission probability (without considering exposure controls) and severity band and provides advice on what engineering controls to use. It includes nine domains covering handling of liquids, powders, and abrasion of solids. Combines hazard "severity" and exposure "probability" scores in a matrix to obtain a level of risk and associated controls out of 4 possible levels of increasing risk and associated controls.	[39–41]	Risk management
Stoffenmanager Nano	Ranks potential health risks from workplace inhalation exposure to MN and proposes effective RMM. It concerns single particles as well as agglomerates or aggregates and applies to MN that meet all of the following criteria: i) particles are not (water) soluble; ii) particles are synthetically produced and not released as unintentional by-product of e.g., incomplete combustion processes; iii) the size of the primary particles is smaller than 100 nm and/or the specific surface area of the nanopowder is larger than 60 m^2/g	[57]	Risk management
ANSES Nano	The ANSES CB nanotool was developed by the French Agency for Food, Environmental and Occupational Health & Safety (ANSES) to be applied for conducting risk assessment and risk management of work with manufactured nanomaterials or nano-enabled products in industrial settings.	[47,48]	Risk management
Precautionary Matrix for Synthetic Nanomaterials (Swiss Precautionary Matrix)	This tool helps to determine if exposure needs to be controlled, providing advice on whether a precautionary approach is required under normal working conditions, in the worst-case scenario and for the environment.	[42]	Risk management
NanoSafer	NanoSafer is a combined control-banding and risk management tool that enables assessment of the risk level and recommended exposure control associated with production and use of manufactured nanomaterials (e.g., nanoparticles, nanoflakes, nanofibers, and nanotubes) in specific work scenarios. In addition to manufactured nanomaterials, the tool can also be used to assess and manage emissions from nanoparticle-forming processes.	[51]	Risk management
NanoRiskCat	A screening tool that can identify, categorize and rank exposures and effects of nanomaterials used in consumer products based on data available in the peer-reviewed scientific literature and other regulatory relevant sources of information and data. The primary focus was on nanomaterials relevant for professional end users and consumers as, as well as nanomaterials released into the environment. The wider goal of NanoRiskCat is to help manufacturers, downstream end users, regulators, and other stakeholders to evaluate, rank and communicate the potential for exposure and effects through a tiered approach in which the specific applications of a given nanomaterial are evaluated.	[37,38]	Risk management
A low-cost/evidence-based tool	A low-cost/evidence-based for assessing and managing the risks associated with exposure to Carbon Nanofiber	[86]	Risk management
XL Insurance Database	An assessment strategy based on the protocol that XL Insurance uses for calculating insurance premiums for chemical industries. The protocol is mainly used to perform risk assessment for the manufacture of nanomaterials, by focusing on the characteristics of the materials and production processes.	[87,88]	Risk management / Insurance sector
Nano-specific Risk Management Library	The main purpose of the tool is to provide small and medium sized enterprises (SMEs), large companies, and other relevant stakeholders with an easy to use tool to select proper measures to achieve a high level of protection of the human health and the environment against ENMs, assisting them in the selection of adequate personal protective equipment (PPE) and engineering controls (EC) in order to prevent exposure to ENMs and release in the workplace.	RIVM	Risk management

Table S9. Safety-by-design-monitoring tools descriptions and references.

Tool name	Description	References	Sector
ProSafe SbD Implementation Concept	SbD implementation concept based on the NANoREG SbD concept. The four main elements are:<br>#1 The workflows in industrial innovation processes or actor-specific needs<br>#2 The Safety Dossier<br>#3 The Safety Profile<br>#4 Harmonized inventory of SbD protocols, procedures, and data	[89]	SbD
CENARIOS	CENARIOS, the first certifiable nano-specific risk management and monitoring system. CENARIOS provides a "State-of-the Art" hazard and risk assessment, encompassing risk monitoring tools to minimize the potential risks.	[90]	Monitoring

References

1. Hristozov, D.; Gottardo, S.; Semenzin, E.; Oomen, A.; Bos, P.; Peijnenburg, W.; Tongeren, M.; Nowack, B.; Hunt, N.; Brunelli, A.; et al. Frameworks and tools for risk assessment of manufactured nanomaterials. Environ. Int. 2016, 95, 36–53.
2. Hristozov, D.; Gottardo, S.; Critto, A.; Marcomini, A. Risk assessment of engineered nanomaterials: A review of available data and approaches from a regulatory perspective. Nanotoxicology 2012, 6, 880–898.
3. Kasperson, R. Four questions for risk communication. J. Risk Res. 2014, 17, 1233–1239.
4. Stone, V.; Pozzi-Mucelli, S.; Tran, L.; Aschberger, K.; Sabella, S.; Vogel, U.; Poland, C.; Balharry, D.; Fernandes, T.; Gottardo, S.; et al. ITS-NANO—Prioritising nanosafety research to develop a stakeholder driven intelligent testing strategy. Part. Fibre Toxicol. 2014, 11, 9.
5. Hristozov, D.; Zabeo, A.; Alstrup Jensen, K.; Gottardo, S.; Isigonis, P.; Maccalman, L.; Critto, A.; Marcomini, A. Demonstration of a modelling-based multi-criteria decision analysis procedure for prioritisation of occupational risks from manufactured nanomaterials. Nanotoxicology 2016, 10, 1215–1228.
6. Rowe, G.; Frewer, L. Public Participation Methods: A Framework for Evaluation. Sci. Technol. Hum. Values 2000, 25, 3–29.
7. Subramanian, V.; Semenzin, E.; Hristozov, D.; Zondervan-van den Beuken, E.; Linkov, I.; Marcomini, A. Review of decision analytic tools for sustainable nanotechnology. Environ. Syst. Decis. 2015, 35, 29, doi:10.1007/s10669-015-9541-x.
8. Grieger, K.; Linkov, I.; Hansen, S.; Baun, A. Environmental risk analysis for nanomaterials: Review and evaluation of frameworks. Nanotoxicology 2012, 6, 196–212.
9. Bos, P.; Gottardo, S.; Scott-Fordsmand, J.; van Tongeren, M.; Semenzin, E.; Fernandes, T.; Hristozov, D.; Hund-Rinke, K.; Hunt, N.; Irfan, M.A.; et al. The MARINA Risk Assessment Strategy: A Flexible Strategy for Efficient Information Collection and Risk Assessment of Nanomaterials. Int. J. Environ. Res. Public Health 2015, 12, 15007–15021.
10. Grobe, A.; Schneider, C.; Rekic, M.; Schetula, V. Nanotechnologien: Was Verbraucher Wissen Wollen; German Federal Association for Consumer Protection (vzbv): Berlin, Germany, 2008.
11. Weinstein, N.; Sandman, P. Some Criteria for Evaluating Risk Messages. Risk Anal. 1993, 13, 103–114.
12. Aven, T.; Renn, O. Risk Management and Governance Concepts, Guidelines and Applications; Springer: Berlin, Germany, 2010; doi:10.1007/978-3-642-13926-0.
13. Noorlander, C.; Bekker, C.; Soeteman-Hernandez, L.; Sabella, S.; Quik, J.; Peijnenburg, W.; Prina-Mello, A.; Sips, A. NANoREG Deliverable D6.4: Inventory of Existing Regulatory Accepted Toxicity Tests Applicable for Safety Screening of MNMs; RIVM: Bilthoven, The Netherlands, 2016.
14. OECD. Preliminary Analysis of Exposure Measurement and Exposure Mitigation in Occupational Settings: Manufactured Nanomaterials; OECD (Organisation for Economic Cooperation and Development) Series on the Safety on Manufactured Nanomaterials, No. 8; Environmental Directorate Joint Meeting of the Chemical Committee and the Working Party; OECD: Paris, France, 2009.
15. Brouwer, D. Control Banding Approaches for Nanomaterials. Ann. Occup. Hyg. 2012, 56, 506–514.
16. Oberdörster, G.; Oberdörster, E.; Oberdörster, J. Nanotoxicology: An emerging discipline evolving from studies of ultrafine particles. Environ. Health Perspect. 2005, 113, 823–839.
17. RCEP (Royal Commission on Environmental Pollution). Novel Materials in the Environment: The Case of Nanotechnology. The Stationery Office (TSO): Norwich, UK, 2008.
18. WBGU (German Advisory Council on Global Change). World in transition: Strategies for managing global environmental risks (Annual Report); Springer: Heidelberg, Germany, 2000.
19. IRGC. Risk Governance of Nanotechnology Applications in Food and Cosmetics; Grobe, A., Renn, O., Jaeger, A., Eds.; International Risk Governance Council (IRGC): Geneva, Switzerland, 2008.
20. NanoRiskFramework. Environmental defense: DuPont Nano partnership. 2007. Available online: http://www.edf.org/documents/6496_Nano%20Risk%20Framework.pdf (accessed on 24 April 2018).
21. European Commission: Code. Commission Recommendation on a Code of Conduct for Responsible Nanosciences and Nanotechnologies Research. 2008. Available online: https://ec.europa.eu/research/industrial_technologies/pdf/policy/nanocode-rec_pe0894c_en.pdf (accessed on 24 April 2018).

22. IRGC. Risk Governance: Towards an Integrative Approach; White Paper No. 1, O. Renn with An Annex; Graham, P., Ed.; International Risk Governance Council (IRGC): Geneva, Switzerland, 2005.
23. Smita, S.; Gupta, S.; Bartonova, A.; Dusinska, M.; Gutleb, A.; Rahman, Q. Nanoparticles in the environment: Assessment using the causal diagram approach. Environ. Health 2012, 11 (Suppl. 1), 13.
24. Jovanovic, A.; Debray, B.; Ølsen, K.; Balos, D.; Batista, M.; Brouwer, D.H.; Dien, Y.; Dolinski, K.; Duval, C.; Lopez de Ipina, M.J.; et al. Managing Emerging Technology-Related Risks; CWA 16649; CEN: Brussels, Belgium, 2013.
25. Raban, Y.; Remes, M.; Schroder, S. Final Synthesis Report on Security Oriented Foresight Mapping of Outputs and Methods—Deliverable D3.4; 2015.
26. EU CORDIS. Final Report Summary—FORCE (FOResight Coordination for Europe); EU: Luxembourg, 2016.
27. Habegger, B. Horizon Scanning in Government: Concept, Country Experience, and Models for Switzerland; Center for Security Studies: ETH Zurich, Switzerland, 2009.
28. Garnett, K.; Lickorish, F.; Rocks, S.; Prpich, G.; Rathe, A.; Pollard, S. Integrating horizon scanning and strategic risk prioritisation using a weight of evidence framework to inform policy decisions. Sci. Total Environ. 2016, 560–561, 82–91.
29. Swiss RE. Swiss Re's SONAR: New Emerging Risk Insight; Swiss RE: Zürich, Switzerland, 2016.
30. Allianz Risk Pulse. Allianz Risk Barometer. 2017. Available online: https://www.agcs.allianz.com/content/dam/onemarketing/agcs/agcs/reports/Allianz-Risk-Barometer-2017.pdf (accessed on 25 January 2019)
31. UK GOVT. Futurescaper Platform; Digital Marketplace: UK, 2017.
32. Hristozov, D.; Zabeo, A.; Foran, C.; Isigonis, P.; Critto, A.; Marcomini, A.; Linkov, I. A weight of evidence approach for hazard screening of engineered nanomaterials. Nanotoxicology 2014, 8, 72–87.
33. Hristozov, D.; Gottardo, S.; Cinelli, M.; Isigonis, P.; Zabeo, A.; Critto, A.; van Tongeren, M.; Tran, L.; Marcomini, A. Application of a quantitative weight of evidence approach for ranking and prioritising occupational exposure scenarios for titanium dioxide and carbon nanomaterials. Nanotoxicology 2013, 8, 117–131.
34. Grieger, K.; Redmon, J.; Money, E.; Widder, M.; van der Schalie, W.; Beaulieu, S.; Womack, D. A relative ranking approach for nano-enabled applications to improve risk-based decision making: A case study of Army materiel. Environ. Syst. Decis. 2015, 35, 42–53.
35. Tervonen, T.; Linkov, I.; Figueira, J.; Steevens, J.; Chappell, M.; Merad, M. Risk-based classification system of nanomaterials. J. Nanopart. Res. 2009, 11, 757–766.
36. Beaudrie, C.; Kandlikar, M.; Gregory, R.; Long, G.; Wilson, T. Nanomaterial risk screening: A structured approach to aid decision making under uncertainty. Environ. Syst. Decis. 2015, 35, 88–109.
37. Hansen, S.; Baun, A.; Alstrup-Jensen, K. NanoRiskCat—A Conceptual Decision Support Tool for Nanomaterials; Danish Ministry of the Environment: Copenhagen, Denmark, 2011.
38. Hansen, S.; Jensen, K.; Baun, A. NanoRiskCat: A conceptual tool for categorization and communication of exposure potentials and hazards of nanomaterials in consumer products. J. Nanopart. Res. 2014, 16, 2195.
39. Paik, S.; Zalk, D.; Swuste, P. Application of a Pilot Control Banding Tool for Risk Level Assessment and Control of Nanoparticle Exposures. Ann. Occup. Hyg. 2008, 52, 419–428.
40. Murashov, V.; Howard, J. Essential features for proactive risk management. Nat. Nanotechnol. 2009, 4, 467–470.
41. Zalk, D.; Paik, S.; Swuste, P. Evaluating the Control Banding Nanotool: A qualitative risk assessment method for controlling nanoparticle exposures. J. Nanopart. Res. 2009, 11, 1685–1704.
42. Höck, J.; Behra, R.; Bergamin, L.; Bourqui-Pittet, M.; Bosshard, C.; Epprecht, T.; Furrer, V.; Frey, S.; Gautschi, M.; Hofmann, H.; et al. Guidelines on the Precautionary Matrix for Synthetic Nanomaterials; Version 3.1.; Federal Office of Public Health and Federal Office for the Environment: Berne, Switzerland, 2018.
43. Askham, C. Environmental Product Development Combining the Life Cycle Perspective with Chemical Hazard Information. Ph.D. Thesis, Aalborg University, Aalborg, Denmark, 2011.
44. Askham, C.; Gade, A.; Hanssen, O. Combining REACH, environmental and economic performance indicators for strategic sustainable product development. J. Clean. Prod. 2012, 35, 71–78.

45. Askham, C.; Gade, A.; Hanssen, O. Linking chemical risk information with life cycle assessment in product development. J. Clean. Prod. 2013, 51, 196–204.
46. Collier, Z.A.; Kennedy, A.J.; Poda, A.R.; Cuddy, M.F.; Moser, R.D.; MacCuspie, R.I.; Harmon, A.; Plourde, K.; Haines, C.D.; Steevens, J.A. Tiered guidance for risk-informed environmental health and safety testing of nanotechnologies. J. Nanopart. Res. 2015, 17, 155.
47. Ostiguy, C.; Riediker, M.; Triolet, J.; Troisfontaines, P.; Vernez, D. Development of A Specific Control Banding Tool for Nanomaterials; French Agency for Food, Environmental and Occupational Health and Safety (ANSES): Maisons-Alfort, France, 2010.
48. Riediker, M.; Ostiguy, C.; Triolet, J.; Troisfontaine, P.; Vernez, D.; Bourdel, G.; Thieriet, N.; Cadène, A. Development of a Control Banding Tool for Nanomaterials. J. Nanomater. 2012, 2012, 1–8.
49. Subramanian, V.; Semenzin, E.; Hristozov, D.; Zabeo, A.; Malsch, I.; McAlea, E.; Murphy, F.; Mullins, M.; van Harmelen, T.; Ligthart, T.; et al. Sustainable nanotechnology decision support system: Bridging risk management, sustainable innovation and risk governance. J. Nanopart. Res. 2016, 18, 89.
50. Hristozov, D.; Pizzol, L.; Basei, G.; Zabeo, A.; Mackevica, A.; Foss Hansen, S.; Gosens, I.; Cassee, F.R.; de Jong, W.; Koivisto, A.J.; et al. Quantitative human health risk assessment along the lifecycle of nano-scale copper-based wood preservatives. Nanotoxicology 2018, 12, 747–765, doi:10.1080/17435390.2018.1472314.
51. Jensen, K.; Saber, A.; Kristensen, H.; Liguori, B.; Jensen, A.; Koponen, I.; Wallin, H. NanoSafer vs. 1.1 Nanomaterial risk assessment using first order modeling. In Proceedings of the 6th International Symposium on Nanotechnology, Occupational and Environmental Health, Nagoya, Japan, 28–31 October 2013.
52. Park, M.; Catalán, J.; Ferraz, N.; Cabellos, J.; Vanhauten, R.; Vázquez-Campos, S.; Janer, G. Development of a systematic method to assess similarity between nanomaterials for human hazard evaluation purposes—lessons learnt. Nanotoxicology 2018, 12, 1–25.
53. ECETOC. Targeted Risk Assessment (TRA). 2017. Available online: http://www.ecetoc.org/tools/targeted-risk-assessment-tra/ (accessed on 18 January 2019).
54. van Harmelen, T.; Zondervan-van den Beuken, E.; Brouwer, D.; Kuijpers, E.; Fransman, W.; Buist, H.; Ligthart, T.; Hincapié, I.; Hischier, R.; Linkov, I.; et al. LICARA nanoSCAN—A tool for the self-assessment of benefits and risks of nanoproducts. Environ. Int. 2016, 91, 150–160.
55. Zaleski, R.; Qian, H.; Zelenka, M.; George-Ares, A.; Money, C. European solvent industry group generic exposure scenario risk and exposure tool. J. Expo. Sci. Environ. Epidemiol. 2014, 24, 27–35.
56. Koch, W.; Behnke, W.; Berger-Preiß, E.; Kock, H.; Gerling, S.; Hahn, S.; Schröder, K. BAuA—Repositorium—Validation of An EDP Assisted Model for Assessing Inhalation Exposure and Dermal Exposure during Spraying Processes—Bundesanstalt für Arbeitsschutz und Arbeitsmedizin; BAUA: Dresden, Germany, 2012.
57. Van Duuren-Stuurman, B.; Vink, S.; Verbist, K.; Heussen, H.; Brouwer, D.; Kroese, D.; Van Niftrik, M.F.J.; Tielemans, E.; Fransman, W. Stoffenmanager Nano Version 1.0: A Web-Based Tool for Risk Prioritization of Airborne Manufactured Nano Objects. Ann. Occup. Hyg. 2012, 56, 525–541.
58. Meesters, J.; Koelmans, A.; Quik, J.; Hendriks, A.; van de Meent, D. Multimedia Modeling of Engineered Nanoparticles with SimpleBox4nano: Model Definition and Evaluation. Environ. Sci. Technol. 2014, 48, 5726–5736.
59. Quik, J.; de Klein, J.; Koelmans, A. Spatially explicit fate modelling of nanomaterials in natural waters. Water Res. 2015, 80, 200–208.
60. De Klein, J.; Quik, J.; Bäuerlein, P.; Koelmans, A. Towards validation of the NanoDUFLOW nanoparticle fate model for the river Dommel, The Netherlands. Environ. Sci. Nano 2016, 3, 434–441.
61. Liu, H.; Cohen, Y. Multimedia Environmental Distribution of Engineered Nanomaterials. Environ. Sci. Technol. 2014, 48, 3281–3292.
62. Liu, H.; Bilal, M.; Lazareva, A.; Keller, A.; Cohen, Y. Simulation tool for assessing the release and environmental distribution of nanomaterials. Beilstein J. Nanotechnol. 2015, 6, 938–951.
63. Dumont, E.; Williams, R.; Keller, V.; Voß, A.; Tattari, S. Modelling indicators of water security, water pollution and aquatic biodiversity in Europe. Hydrol. Sci. J. 2012, 57, 1378–1403.

64. Dumont, E.; Johnson, A.; Keller, V.; Williams, R. Nano silver and nano zinc-oxide in surface waters—Exposure estimation for Europe at high spatial and temporal resolution. Environ. Pollut. 2015, 196, 341–349.
65. Delmaar, J.E.; van der Zee Park, M.; van Engelen, J.G.M. ConsExpo—Consumer Exposure and Uptake Models—Program Manual; RIVM: Bilthoven, The Netherlands, 2006.
66. Gottschalk, F.; Scholz, R.W.; Nowack, B. Probabilistic material flow modeling for assessing the environmental exposure to compounds: Methodology and an application to engineered nano-TiO2 particles. Environ. Model. Softw. 2010, 25, 320–332, doi:10.1016/j.envsoft.2009.08.011.
67. Gottschalk, F.; Sonderer, T.; Scholz, R.W.; Nowack, B. Possibilities and limitations of modelling environmental exposure to engineered nanomaterials by probabilistic material flow analysis. Environ. Toxicol. Chem. 2010, 29, 1036–1048, doi:10.1002/etc.135.
68. Bornhöft, N.; Sun, T.; Hilty, L.; Nowack, B. A dynamic probabilistic material flow modeling method. Environ. Model. Softw. 2016, 76, 69–80.
69. Boxall, A.B.; Chaudhry, Q.; Sinclair, C.; Jones, A.; Aitken, R.; Jefferson, B.; Watts, C. Current and Future Predicted Environmental Exposure to Engineered Nanoparticles; Central Science Laboratory, Department of the Environment and Rural Affairs: London, UK, 2007.
70. Mueller, N.; Nowack, B. Exposure Modeling of Engineered Nanoparticles in the Environment. Environ. Sci. Technol. 2008, 42, 4447–4453.
71. Arvidsson, R.; Molander, S.; Sandén, B. Impacts of a Silver-Coated Future: Particle Flow Analysis of Silver Nanoparticles. J. Ind. Ecol. 2011, 15, 844–854.
72. Arvidsson, R.; Molander, S.; Sandén, B.; Hassellöv, M. Challenges in Exposure Modeling of Nanoparticles in Aquatic Environments. Hum. Ecol. Risk Assess. Int. J. 2011, 17, 245–262.
73. Riviere, J.E. Pharmacokinetics and Biodistribution of Nanomaterials. In Nanotoxicology: Progress toward Nanomedicine, 2nd ed.; Riviere, J.E., Monteiro-Riviere, N., Tran, C., Vesterdal L., Eds.; CRC Press: Boca Raton, FL, USA, 2014; p. 502.
74. Niesmann, K.; Baron. M. 'Nano to Go'—BAuA's Latest Contribution to NanoValid; BAUA: Dresden, Germany, 2015.
75. Anjilvel, S.; Asgharian, B. A Multiple-Path Model of Particle Deposition in the Rat Lung. Fundamental and Appl. Toxicol. 1995, 28, 41–50.
76. RIVM. Multiple Path Particle Dosimetry Model (MPPD v 1.0): A Model for Human and Rat Airway Particle Dosimetry; RIVM: Bilthoven, The Netherlands, 2002.
77. Asbach, C.; Kuhlbusch, T.A.J.; Kaminski, H.; Stahlmecke, B.; Plitzko, S.; Götz, U.; Dahmann, D. Standard Operation Procedures for assessing exposure to nanomaterials, following a tiered approach. Nano Gem 2012, 1-90.
78. Jeliazkova, N.; Chomenidis, C.; Doganis, P.; Fadeel, B.; Grafström, R.; Hardy, B.; Hastings, J.; Hegi, M.; Jeliazkov, V.; Kochev, N.; et al. The eNanoMapper database for nanomaterial safety information. Beilstein J. Nanotechnol. 2015, 6, 1609–1634.
79. Marvin, H.; Bouwmeester, H.; Bakker, M.; Kroese, E.; van de Meent, D.; Bourgeois, F.; Lokers, R.; van der Ham, H.; Verhelst, L. Exploring the development of a decision support system (DSS) to prioritize engineered nanoparticles for risk assessment. J. Nanopart. Res. 2013, 15, 1839.
80. Gottschalk, F.; Nowack, B. A probabilistic method for species sensitivity distributions taking into account the inherent uncertainty and variability of effects to estimate environmental risk. Integr. Environ. Assess. Manag. 2013, 9, 79–86.
81. Semenzin, E.; Lanzellotto, E.; Hristozov, D.; Critto, A.; Zabeo, A.; Giubilato, E.; Marcomini, A. Species sensitivity weighted distribution for ecological risk assessment of engineered nanomaterials: The n-TiO2 case study. Environ. Toxicol. Chem. 2015, 34, 2644–2659.
82. Safe Work Australia. Work Health and Safety Assessment Tool for Handling Engineered Nanomaterials; Safe Work Australia: Canberra, Australia, 2010.
83. Money, E.; Reckhow, K.; Wiesner, M. The use of Bayesian networks for nanoparticle risk forecasting: Model formulation and baseline evaluation. Sci. Total Environ. 2012, 426, 436–445.
84. Money, E.; Barton, L.; Dawson, J.; Reckhow, K.; Wiesner, M. Validation and sensitivity of the FINE Bayesian network for forecasting aquatic exposure to nano-silver. Sci. Total Environ. 2014, 473–474, 685–691.

85. Reihlen, A.; Jepsen, D. Discussion and Results of the German NanoCommission's Work and the Stakeholder Dialogue "Risk Management in the Nano World"; German Federal Ministry of the Environment, Nature Conservation and Nuclear Safety: Hamburg, Germany, 2012.
86. Genaidy, A.; Sequeira, R.; Rinder, M.; A-Rehim, A. Risk analysis and protection measures in a carbon nanofiber manufacturing enterprise: An exploratory investigation. Sci. Total Environ. 2009, 407, 5825–5838.
87. Sellers, K. Balancing the Risks and Rewards. In Nanotechnology and the Environment; Sellers, K., Sellers, J.K., Mackay, C., Bergeson, L.L., Clough, S.R., Hoyt, M., Chen, J., Henry, K., Eds.; CRC Press: Boca Raton, FL, USA, 2009; p. 281.
88. Robichaud, C.O.; Tanzil, D.; Weilenmann, U.; Wiesner, R.M. Relative Risk Analysis of Several Manufactured Nanomaterials: An Insurance Industry Context. Environ. Sci. Technol. 2005, 39, 8985–8994, doi:10.1021/es0506509.
89. TEMAS & IPC. ProSafe Safe-by-Design (SbD) Implementation Concept Final; RIVM: Bilthoven, The Netherlands, 2016.
90. Widler, T.; Meili, C.; Semenzin, E.; Subramanian, V.; Zabeo, A.; Hristozov, D.; Marcomini, A. Organisational Risk Management of Nanomaterials Using SUNDS: The Contribution of CENARIOS®; Widler, T., Meili, C., Semenzin, E., Subramanian, V., Zabeo, A., Hristozov, D., Marcomini, A., Eds.; Springer: Cham, Switzerland, 2016.
